# Orthodoxy, *illusio,* and playing the scientific game: a Bourdieusian analysis of infection control science in the COVID-19 pandemic

**DOI:** 10.12688/wellcomeopenres.16855.2

**Published:** 2021-09-22

**Authors:** Trisha Greenhalgh, Mustafa Ozbilgin, Damien Contandriopoulos

**Affiliations:** 1 Nuffield Department of Primary Care Health Sciences, University of Oxford, Oxford, OX2 6GG, UK; 2 Brunel University London, Uxbridge, UB8 3PH, UK; 3 School of Nursing, University of Victoria, Victoria, British Colombia, V8P 5C2, Canada

**Keywords:** SARS-CoV-2, aerosol transmission, Bourdieu, illusio, orthodoxy, symbolic violence, evidence-based medicine, infection prevention and control

## Abstract

Background:

Scientific and policy bodies’ failure to acknowledge and act on the evidence base for airborne transmission of SARS-CoV-2 in a timely way is both a mystery and a scandal. In this study, we applied theories from Bourdieu to address the question, “How was a partial and partisan scientific account of SARS-CoV-2 transmission constructed and maintained, leading to widespread imposition of infection control policies which de-emphasised airborne transmission?”.

Methods:

From one international case study (the World Health Organisation) and four national ones (UK, Canada, USA and Japan), we selected a purposive sample of publicly available texts including scientific evidence summaries, guidelines, policy documents, public announcements, and social media postings. To analyse these, we applied Bourdieusian concepts of field, *doxa*, scientific capital, *illusio,* and game-playing. We explored in particular the links between scientific capital, vested interests, and policy influence.

Results:

Three fields—political, state (policy and regulatory), and scientific—were particularly relevant to our analysis. Political and policy actors at international, national, and regional level aligned—predominantly though not invariably—with medical scientific orthodoxy which promoted the droplet theory of transmission and considered aerosol transmission unproven or of doubtful relevance. This dominant scientific sub-field centred around the clinical discipline of infectious disease control, in which leading actors were hospital clinicians aligned with the evidence-based medicine movement. Aerosol scientists—typically, chemists, and engineers—representing the heterodoxy were systematically excluded from key decision-making networks and committees. Dominant discourses defined these scientists’ ideas and methodologies as weak, their empirical findings as untrustworthy or insignificant, and their contributions to debate as unhelpful.

Conclusion:

The hegemonic grip of medical infection control discourse remains strong. Exit from the pandemic depends on science and policy finding a way to renegotiate what Bourdieu called the ‘rules of the scientific game’—what counts as evidence, quality, and rigour.

## Introduction

### The droplet v aerosol debate

*“A good scientist is someone who has a sense of the scientific game”*

                                                                                                                                                                                                                                       – Pierre Bourdieu (page 83) ^1^

When the World Health Organisation (WHO) declared COVID-19 a “pandemic” on 11 ^th^ March 2020, the virus had already caused 100,000 known cases and 4,000 deaths in 114 countries. The WHO had summarised its recommendations for reducing spread of the disease in a widely-disseminated 90-second video animation released on 28 ^th^ February 2020, entitled ‘How is COVID-19 spread and how do you protect yourself against it? *’* (source A4, Table 1). Its full text was as follows (emphasis added):

*“COVID-19 is an infectious disease caused by a new coronavirus introduced to humans for the first time. It is spread from person to person mainly through the droplets produced when an infected person speaks, coughs or sneezes. These droplets can land in the mouths or noses of people who are nearby. These droplets are too heavy to travel far in the air – they only travel approximately one metre and quickly settle on surfaces. This is the reason person-to-person spread is happening between close contacts. The exact time that the virus can survive on surfaces is not yet known. So it is wise to clean surfaces regularly, particularly in the vicinity of people infected with COVID-19. Hands touch many surfaces, which can be contaminated with the virus. You should therefore avoid touching your eyes, nose or mouth, since contaminated hands can transfer the virus from the surface to yourself. When coughing or sneezing, cover your mouth and nose with the bend of your elbow or use a disposable tissue. If a tissue is used, discard it immediately into a closed bin. The most effective way to prevent the spread of the new coronavirus is to wash hands frequently with an alcohol-based hand rub or soap and water. This will eliminate the virus if it is on your hands. Stay healthy and prevent the spread of COVID-19.”* (World Health Organisation, 28 ^th^ February 2020; Source A4, Table 1)

The underlined sections in the above quote, which reflect a droplet mode of transmission, are scientifically questionable (and in our view incorrect). The SARS-CoV-2 virus can travel long distances in the air where it remains viable for up to eight hours; spread is not always ‘person to person’; the virus enters the body mainly by inhalation via the lungs; handwashing and surface cleansing are relatively minor ways of preventing spread; and airborne spread is the main mechanism of transmission even within a one-metre distance ^2– 5^. If the virus was spread predominantly through large droplets, prevention should focus on reducing direct contact, cleaning surfaces, physical barriers (such as plastic screens), physical distancing (e.g., two metres or six feet) and masking within that distance, and high-grade protection for healthcare staff when conducting so-called ‘aerosol-generating medical procedures’ (AGMPs). But given that transmission is predominantly airborne, different measures are needed including ventilation, air filtration, reducing crowding and time spent indoors, greater attention to the quality and fit of masks, more widespread masking when indoors, and extensive higher-grade protection for healthcare and other at-risk staff. Measures to counter aerosol transmission are more difficult, more costly in the short term, and (therefore) politically less popular.

We acknowledge that not all scientists accept the statements in the previous paragraph – see for example ^6, 7^. Indeed, non-acceptance of the theory of airborne spread is the central focus of this paper. Our aim is not to contribute to the scientific stand-off between ‘droplet’ and ‘airborne’ theories (we have done that elsewhere ^2, 8^), but to analyse why, in the face of considerable evidence in support of airborne spread, this stand-off is happening and why theories of transmission which dismiss the aerosol route continue to dominate policy in many settings.

We write (in autumn 2021) with a sense of urgency, at a time when pandemic fatigue is palpable but hundreds of thousands of new cases of COVID-19 are occurring daily. Children are returning to face-to-face learning in contexts which often emphasise droplet—and, to a lesser extent, fomite—precautions (especially physical distancing, separation of learners by plastic screens, and handwashing) over airborne ones (such as opening windows, masking in classrooms, and reducing time spent indoors) ^9, 10^. Droplet and fomite theories also dominate in healthcare facilities, where the emphasis remains on physical distancing, cleaning surfaces, and handwashing ^11^, and where work (with few exceptions) is divided into formally-designated AGMPs, such as intubation, for which higher-grade personal protective equipment (PPE) is needed and all other tasks (non-AGMPs), in which, it is assumed, no significant aerosols are generated ^12^. Whereas AGMPs are mostly undertaken by senior doctors, more junior staff who attend to coughing and breathless patients in poorly ventilated spaces can rarely access high-grade PPE.

The arguments about the importance of aerosol transmission, therefore, could not be more pressing or more politically charged. Against this background, our research questions are as follows:

- How have the symbolic and hegemonic struggles for power and influence among two subfields of health sciences, one of which promotes traditional contact-and-droplet infection control precautions and the other that promotes aerosol precautions, shaped the regulation and practice of infection control at international, national, and local level?
- How did these positions of influence arise – and how are they maintained?
- What would need to change in order for heterodox science of critical import, i.e., from aerosol scientists, to exert more influence on policy?

In the next sections, we introduce our theoretical perspective, methodology, and dataset before describing the international policy context and four national or regional case studies. In the analysis, we present our Bourdieusian theorisation of the contribution of two competing scientific sub-fields to a fast-moving, politically- and ideologically-charged crisis situation, highlighting not just their epistemological assumptions and preferred methodologies but also (in one case) the field’s hard-won and fiercely-defended hegemonic power and (in the other) its drawn-out struggle for legitimacy and influence. In the discussion, we offer some theoretically driven possibilities for a difficult but mission-critical renegotiation of the ‘rules of the game’. 

### Theoretical approach

We use Bourdieu’s linked concepts of fields, *doxa*, capital, and *illusio* to understand how individuals and institutions draw on varied forms of resources, and a range of beliefs and assumptions, in order to navigate their relations and positions and achieve their interests.

For Bourdieu, the social world is highly political. It is shaped by agents’ involvement in struggles to impose their views on and wield their power over others. But those struggles are not cynical games played by rational actors, nor are they the deterministic effects of structural forces on passive actors. By virtue of their habitus (durable, transposable dispositions which structure how they perceive the world and act within it), Bourdieusian agents are both products and creators of their social environments ^13^. The way habitus is formed over time and through repetition accounts for the taken-for-granted practices and deeply-held assumptions among groups of people in specific fields of relations.

Social struggles for the imposition of truth, prestige and resources are located in – and specific to – particular fields (which Bourdieu defined as specific areas of the broader social space ^14, 15^). The relative value of different types of capital (economic, social, cultural and symbolic) as well as internalised rules and vested interests in the game ( *illusio*) both define the field’s boundaries and are shaped by it 16. Practically, being part of a field means having more in common with your foes within that field than with others outside the field. Agents within a field will share compatible assessment of the relative position and prestige of others. Symbolic capital – the clout and prestige one can effectively mobilise –will vary from one field to the next.

The concept of field rests on a tension between, on the one hand, each field's autonomy and specificities and, on the other, its imbrication in a shared social space and relations of power ( *champ du pouvoir*). For a field to be sociologically relevant, it needs to be structured by its agents' common intertwined habitus and *illusio*. But no field is ever entirely disconnected from all the others, because—at the micro level—the same agents are involved in multiple fields and—at the macro level—fields overlap and interconnect.

Bourdieu ^13^ defines the concept of *doxa* as a set of deeply held and taken for granted assumptions among people in a particular context. While some beliefs get established and they are considered as legitimate (orthodoxy), other forms of emergent beliefs remain outside the established order and are considered marginal (heterodox) to it. Each scientific field—and other fields of relations—has orthodoxies and heterodoxies. The orthodoxy retains its control over what counts as rigour and relevance, what is possible, and what is acceptable (in this case scientifically). The heterodoxy challenges the power and influence of orthodoxy on defining the terms of science. Some scientific sub-disciplines are considered more prototypical (normatively idealised as scientific) than others; some are considered typical (conforming to general expectations of science) and some atypical (underrepresented or devalued in relation to other disciplines). Broadly speaking, ‘hard’, ‘pure’, ‘mathematical’, and ‘exact’ sciences are valued and idealised, forming orthodoxies, while ‘soft’, ‘applied’, ‘social’, and ‘inexact’ sciences tend to be less valued in comparison, and relegated often to heterodox positions. Heterodoxy could bring innovation from the margins, if cultivated and better regarded by the leadership ^17^. Heterodox evidence would find better reception in scientific contexts that value interdisciplinarity, multidisciplinarity and transdisciplinarity and recognise the innovative potential of pluralism over purism.

Science capital is a term which Archer *et al.* developed ^18^, based on Bourdieusian term of cultural capital, which refers to the knowledge, education, intellect, insight, skills, and understanding that an individual could deploy to shape their status and power in a particular field. In the case of scientific capital, individuals garner knowledge, skills, and abilities that help them gain status, recognition, power, and access to resources such as research grants and contracts, and to get their scientific knowledge widely used. Inter-field struggles for status and prestige could warp how certain forms of science capital may be recognised and valued while other forms are marginalised. 

### Three fields

The decisions at play lie at the intersection of at least three fields. First, the properly political field of agents vying, mostly through symbolic struggles, for elected positions within the national and regional jurisdictions ^19^ as well as the mediatic ecosystem whose function is to make public the events occurring in that field. For Bourdieu, the political field is not to be confused with the State, despite the control that agents involved in it can have on the State. The political field and the strategies of its agents are structured by political parties and factions, and the quest for prestige and centrality.

Second, the public health policy administration as a function of the state and as a sub-field of the bureaucracy field (a different but overlapping field to academic public health). Bourdieu extends the Weberian notion of the state's monopoly over legitimate physical violence to a capacity to impose its own categories and exert symbolic violence – that is, a type of non-physical violence manifested in the power differential between social groups. The pandemic revealed the powers which the sub-field of public health has to impose physical and symbolic violence, sometimes embedded in specific laws, and brought those to the forefront of daily lives. But the transnational nature of the pandemic also brought international public health bodies (notably the WHO) to play a role in many national symbolic struggles. Notably, the policy-regulatory field is not unified. It also includes competing statutes of various kinds and legal decisions, some of which uphold and others that challenge the status quo.

Finally, the field of science *per se*, which Bourdieu depicted as structured by symbolic struggles for positions, prestige, and so on. Even in sub-fields where the ‘purest’ science (such as particle physics) is produced and reproduced, that science is in some respects a social field like all others—with its relations of force, its powers, its struggles and profits, its generic mechanisms such as those that regulate the selection of newcomers or the competition between the various producers ^14^. Fields and sub-fields help progress specialist science by developing normative standards and supporting greater rigor within those standards—but they also bring a risk of balkanization, with the triple hazards of “monopoly, monotony, and isolation” ^20^. Bourdieu also emphasises that the field is constituted by the common *illusio* needed to perceive the distinction between orthodox and heterodox positions ^14^. Recognition of this distinction is important for scientific progress as heterodox science challenges the very monopoly, monotony and isolation that the scientific orthdoxy upholds.

In sum, a dynamic understanding of struggles of symbolic power and control among politics, State and scientific subfields provides a unique lens through which we can consider the extent to which the evidence of aerosol scientists was not merely overlooked but symbolically degraded by the hegemonic scientific order.

## Methods

The study was conducted between March and April 2021, using a purposive sample of sources including policy documents, public statements, press articles, tweets from organisations, videos, letters, and academic and grey literature publications produced during the pandemic (or, in a few cases, before it). Sources were selected for their contribution to building some hard cases that would help us answer the research questions set out above about how power struggles arose and played out between scientists promoting contact-and-droplet transmission and those promoting aerosol transmission. Initially, we chose to develop three in-depth cases of how the science was interpreted and drawn upon in worker-employer disputes in three different countries, because the stories and issues were prominent in the media at the time and familiar to the authors. As described below, we added a contrasting case and international context as our analysis progressed. Following our approach to the University of Oxford’s Medical Sciences Division Research Ethics Committee, a representative from the University’s Research Services (Ethics and Integrity) Department confirmed that formal ethics approval was unnecessary because the study was desk research, all sources were in the public domain and social media postings from individual accounts were excluded.

With a view to drawing out the Bourdieusian theoretical elements described in the previous section, we sought to study phenomena at two interconnected levels: at an individual level, the dispositions and practices of individual agents (including scientists, policymakers, and front-line workers); and at a more macro level, the external social structures which formed the strategic terrain within which these human agents assessed reality, made choices, and took action.

Bourdieu’s empirical work is essentially focused on two such intersections. First, the intersection between the conceptual apparatus he developed (field, *doxa*, habitus, *illusio*, objectification, symbolic struggle, etc.) and their practical applications to understand day-to-day behaviours and lived experiences. Second, the intersection between the individual and social levels through what Bourdieu called ‘double binds’ between the structuring and the structured structures ^21^.

Many of Bourdieu’s most important contributions ^15, 22, 23^ rest on an in-depth, careful analysis of micro-level data and lived experiences to support a dialogical back and forth between theory and macro-level systematization. We adopted a similar dialogical approach in our own work here, with the acknowledged limitation that without an empirical component the micro-level data available to us were limited and only from secondary sources. Using the techniques of critical social science (close reading, reflexivity, discussion, theorisation), we sought to understand both the behaviour and motivations of individual actors and the various external forces which were driving that behaviour. In the same way, we rely on illustrative cases and micro-level data such as statements, administrative decisions, or events to build a broader social level explanation of the processes at stake.

To allow for detailed close reading and micro-analysis, we selected five small datasets, which served as a window to a much wider set of issues. One – chosen to provide the international policy context – centres on the early (and to some extent, continuing) alignment of WHO policy and guidance with a droplet mode of transmission. The others were chosen to provide contrasting examples of how this WHO guidance was interpreted and actioned at national and regional level. Three case studies centre on different challenges to policymakers from healthcare workers (UK), healthcare workers and classroom teachers (Canada), and a challenge by a supermarket worker to his employer (USA). In these, we began with the small-scale social situation and ‘zoomed out’ to ask what wider and more distant influences were relevant. We were interested primarily in what key actors believed to be scientifically true, right, and reasonable – and why they considered certain facts to be untrue or unimportant and certain courses of action to be inappropriate or unnecessary. A final case study, from Japan (selected from several Asian countries which took similar approaches), illustrates a contrasting policy approach in which airborne spread of the virus was accepted and acted on at an early stage.

The texts and transcripts used to construct these case studies are shown in Table 1, which provides hyperlinks to online sources. Identification of most of these sources occurred through systematic citation-tracking from the first document identified. For example, a tweet or press article might link to a policy announcement which in turn cited a policy document, which referenced some peer-reviewed (or not) research studies. Our dataset was thus built using not technical decisions (e.g., using formal inclusion or exclusion criteria) but hermeneutic ones (the contribution of the source to building a richer picture of the case) ^24^. In each case, we ceased data collection once all authors agreed that the story was sufficiently detailed to allow meaningful theoretical analysis. Importantly, we included all disconfirming data—that is, material which initially appeared to contradict our preliminary interpretations—and used this material to seek out more nuanced explanations. No data source was excluded from the analysis, though for space reasons we have omitted some details.

### Case descriptions

***The World Health Organisation.*** At the WHO’s first international press conference on the new, as-yet unnamed coronavirus on 11 ^th^ February 2020 (source A2, Table 1), its Director-General began by emphasising the importance of handwashing and using paper tissues to catch sneezes. Whilst he went on to state (page 10) that *“corona[virus 19] is airborne”*, he shortly afterwards corrected himself:

*“Okay. Sorry, I used the military word, airborne. It meant to spread via droplets or respiratory transmission. Please take it that way; not the military language. Thank you.”* (source A2, Table 1, page 12)

As its current guidance on the topic (source A1, Table 1 ^25^) states, the term ‘military’ reflects the WHO’s advisory role on biological weapons, in which unknown respiratory pathogens are routinely classified as airborne threats—hence, potentially extremely perilous—until firm evidence allows them to be reclassified.

The corrected message that SARS-CoV-2 was “not airborne” had limited impact initially - public health officials inspecting outbreaks in newly-affected countries donned scarce hazmat suits, for example. In the face of what appeared to be excessive caution by these countries, and in the context of a global shortage of PPE ^26^, the WHO found it necessary to underline its message with a ‘fact check’ Tweet (source A5, Table 1) on the 28 ^th^ March 2020 stating *“COVID-19 is NOT airborne”* to counter what it called ‘fake news’ about potential airborne spread and re-emphasise contact and droplet modes of transmission.

This uncompromising message from the WHO in March 2020 may partly explain why politicians and policymakers in the West rapidly aligned with a droplet theory of transmission and ignored, downplayed, or rejected the work of scientists proposing an airborne route.

Scientific briefings produced by the WHO (e.g., source A11, Table 1) and national policy bodies (see next sections) reveal longstanding errors in the science of bioaerosols which have been perpetrated in derivative publications over the years. In particular, three flawed assumptions, whose origins can be traced to the limitations of scientific equipment in the late 19 ^th^ and early 20 ^th^ centuries ^27^, distorted the thinking of policymakers and the non-expert scientists who were advising them: that there is a clear dichotomy between droplets (above five microns) and aerosols (below five microns); that the former explain transmission of respiratory diseases within two metres whereas the latter account only for transmission beyond that distance; and that transmission dynamics of bioaerosols in coughs, sneezes and exhaled air are linear and predictable ^5, 8^. These three oversimplifications may have been unconsciously seized upon by policymakers through a process of satisficing – that is, in the face of urgency, threat, and uncertainty, ensuring that their decisions made sense and were accountable within a selected range of parameters ^28^. Droplet theory, especially when anything below five microns was incorrectly defined as a droplet (in reality, particles of up to 100 microns can be carried long distances in the air), made possible the individualist *“how to protect yourself…”* message (source A4, Table 1) based on personal cleanliness and a simple physical distancing rule. If these were the key measures needed, the WHO would not have to concern itself with such matters as the chemistry of air composition, the physics of air flow, or the architecture of the built environment; it reduced the risk of mass panic at the idea of uncontrolled spread of a new and poorly-understood disease through the very air we breathe; and it made the worldwide shortage of PPE less urgent.

The WHO’s position on prevention of COVID-19 up to early 2021 was based largely on advice from its Infection Prevention and Control Research and Development Expert Group for COVID-19 (IPCRDEG-C19). Most members of that committee are hospital clinicians with specialist training in infectious diseases; they were also strong adherents to the tenets of evidence-based medicine, which is based on empiricist assumptions and reluctant to consider types of evidence beyond randomised controlled trials, as its briefing document on guideline development attests (source A20, Table 1). As public health professor Raina MacIntyre described in a blog (source A15, Table 1), these committee members are experts in topics such as wound management—for which droplet spread is predominant and handwashing is an effective intervention. A leading member of the IPCRDEG’s Secretariat recently led an international handwashing campaign *“to consistently improve hand hygiene practices as a whole-of-society approach to stop the spread of SARS-CoV-2”* (source A18, Table 1 ^29^).

On 6 ^th^ July 2020, 238 aerosol scientists from around the world published an open letter addressed to international policymaking bodies—among which the WHO was implicitly highlighted—summarising studies undertaken by its signatories which had demonstrated *“beyond any reasonable doubt”* that the SARS-CoV-2 virus is released in particles small enough to be carried long distances in the air when people talk, cough, and even just exhale (source A8, Table 1 ^3^). Three days later, following press coverage of the letter—some somewhat negative (source A9, Table 1 ^30^) and some more positive (source A10, Table 1 ^31^)—the WHO published a new Scientific Brief on Transmission of SARS-CoV-2 (source A11, Table 1 ^32^). This document, which remains current at the time of writing, acknowledged the existence of various aerosol studies but considered those studies flawed in various ways and concluded that *“transmission of SARS-CoV-2 by this type of aerosol route [i.e. coughing, speaking, singing, breathing] has not been demonstrated”* ^31^. 

A few weeks later, members of the IPCRDEG-C19 committee published an article (source A12, Table 1 ^7^) with its Chair as lead author, declaring that *“Multiple clinical and epidemiologic reports have now lent considerable support that the predominant route of human-to-human transmission of the SARS-CoV-2 is through respiratory droplets and/or contact routes and do not support significant airborne transmission”* (7, page 2). The academic sources cited in that paper to substantiate the droplet theory were remarkably sparse: they consisted of a report of person-to-person transmission within a single family ^33^, a single hospital case in which air samples had been negative for the virus ^34^, and a single air flight in which nobody got infected ^35^. A letter to the editor (source A14, Table 1 ^36^) argued that the paper was highly selective in its citation of evidence (e.g., it omitted several studies which had found viable virus in the air) and included some fundamental errors of reasoning (e.g., conflating what the authors took to be lack of evidence in favour of aerosol spread with evidence refuting such spread) ^36^.

Some WHO documents contain a key logical fallacy that dominance of close-contact transmission excludes a major role for aerosols. For example, in its July scientific brief (source A11, Table 1), the WHO states: *“Current evidence suggests that transmission of SARS-CoV-2 occurs primarily between people through direct, indirect, or close contact with infected people through infected secretions such as saliva and respiratory secretions, or through their respiratory droplets”* ^32^. This flawed logic was widely reproduced in national-level scientific briefings. For example, the US Centers for Disease Control and Prevention’s scientific brief from October 2020 states: *“The epidemiology of SARS-CoV-2 indicates that most infections are spread through close contact, not airborne transmission”* ^37^. As noted above, aerosol transmission occurs predominantly at close contact ^5^, so dominance of close-contact transmission cannot be taken as evidence that droplet mode dominates.

Whilst the WHO has shifted its position substantially since the beginning of 2021 (for example, recommending ventilation of indoor spaces alongside handwashing and physical distancing from March 2021 – source A17, Table 1 ^38^), its guidance at the time of writing remains primarily focused on measures to reduce close-range droplet transmission except in the specific circumstances of AGMPs, as its living guideline (source A16, Table 1 ^39^) points out. A living systematic review of the role of airborne transmission commissioned by the WHO and including the Chair of the IPCRDEG-C19 as a co-author was published as a preprint on 24 ^th^ March 2021 (source A19, Table 1 ^6^). It considered that no firm conclusions could be drawn about airborne transmission and observed that *“Among case clusters for which airborne transmission is hypothesised, published detailed investigations cannot rule out that droplet and fomite transmission could also explain human-to-human transmission.”* (6, page 4). Notably, neither the living guideline nor the living systematic review included any aerosol scientists as co-authors. 

The members of the WHO’s IPCRDEG had impressive credentials in the fields of both medical science and national policymaking. Its Chair, for example, is a Professor of Medicine and past President of the Canadian Infectious Disease Society, past Board Chairman of the Canadian Committee on Antibiotic Resistance, and a recipient of a Distinguished Service Medal from the Alberta Medical Association and the Order of Canada for Services to Medicine (source A21, Table 1). A Bourdieusian analytic lens would observe that the higher an individual’s endowment of capitals, the stronger the stakes in a game they would have. In such a high-stakes game, highly endowed become the custodians of power, privilege, and boundary conditions of the game. Given their position of seniority and legitimacy within the field and the associated capitals, it would be difficult for them to challenge the rules of the game (the *illusio*) from within and to accept knowledge from the margins.

***United Kingdom.*** Our UK example centres around an open letter sent to the UK Prime Minister on 19 ^th^ February 2021, led by the Royal College of Nursing and signed by 18 other healthcare workers’ organisations including paramedics, podiatrists, nutritionists, speech and language therapists, and porters (source B3, Table 1 ^40^). The authors asked for better ventilation and higher-grade PPE across a wide range of healthcare settings and extending beyond AGMPs.

The open letter challenged the UK’s current COVID-19 Infection Prevention and Control (IPC) Guidance (source B1, Table 1 ^41^), updated in January 2021, which restricted higher-grade PPE to staff—mostly senior doctors—performing AGMPs. It also criticised a state-commissioned ‘rapid review’ document published by the Antimicrobial Resistance and Healthcare Associated Infection (ARHAI) (source B2, Table 1 ^42^), on which the IPC guidance was based. The ARHAI review was labelled ‘Version 11.0’; it concluded (as the previous 10 versions had done) that the predominant mode of transmission was large droplets at short range and that there was “ *no clear evidence*” (page 9) for airborne spread outside AGMPs; a version 12 published a few weeks later contains the same claims.

An independent evidence assessment commissioned by the Royal College of Nursing (source B4, Table 1 ^43^) described the ARHAI rapid review as flawed and outdated. Front-line nurses gave media interviews describing their concerns about working with minimal protection when patients were unwell and coughing, backed up by aerosol scientists who agreed that coughing would generate virus-laden aerosols for which standard masks were inadequate protection. In one such interview, the presenter reminded the audience that Health Protection Scotland’s latest advice is that there is no evidence to support a change in recommendations and that the Health Secretary had said they would be '‘guided by the experts’ (a reference to infectious diseases doctors). She is quoted:

*We take that really seriously and have adapted the PPE that we provide. But we also have a situation where individual members of staff in NHS or in care are able to exercise their own professional judgement about the circumstance that they face and whether or not they believe that they need additional PPE to that that is currently clinically recommended* (Scottish Health Secretary, quoted on Good Morning Scotland, 2 ^nd^ March 2021 – source B5, Table 1)

We consider this deflection to ‘own professional judgement’ further in our analysis section.

Also noteworthy in this case is the publication on 5 ^th^ March 2021 of new Public Health England guidance on ventilation of indoor spaces (source B6, Table 1 ^44^). In an exact mirror of the WHO—and citing the WHO’s new roadmap on ventilation, published a few days earlier (source A17, Table 1 ^38^)—this new policy document appeared to base its recommendations on an assumed airborne route of transmission but was not accompanied by substantial changes to its other policy documents.

A key player in this case study is the instigator and lead author of the letter to the Prime Minister, the Professional Lead of the Infection Prevention and Control Network at the Royal College of Nursing (RCN) (source A22, Table 1). She is a registered nurse, with many years’ clinical experience along with a two-year secondment managing professional standards at the RCN. The Professional Lead role requires her to engage with front-line nurses and ensure the highest standards of infection control in their clinical work. She is the person within the nurses’ professional body to whom registered nurses would direct their concerns about unsafe practices. As the letter she drafted (source B3, Table 1 ^40^) illustrates, she appears to feel a strong sense of moral responsibility to prevent further deaths of frontline healthcare workers.

***Canada.*** In this case study, we focus on guidance relating to schools in one Canadian province, British Columbia, and a legal challenge by healthcare workers in another province, Quebec.

From the very beginning of the pandemic, British Columbia based its prevention measures on an explicit contact, droplet, and fomite theory of transmission. A tweet posted by from British Columbia’s Centre for Disease Control (source C1, Table 1) on 11 ^th^ February 2020, for example, linked to a video by a physician epidemiologist and stated: *“The new #coronavirus is spread by droplets that come from the mouth or nose. The droplets don’t stay floating in the air. This is not an airborne virus.”*

Many interventions, such as the state-wide policy of closing children’s playgrounds and disabling traffic light push buttons, revealed a prevailing fear of droplet and fomite transmission. But as evidence on airborne transmission accumulated during the late spring and summer of 2020, pressure from workers’ unions, supported by aerosol scientist researchers, mounted for the province to adopt measures to reduce airborne spread.

However, the province authorities long resisted the idea of airborne transmission. British Columbia’s Provincial Health Officer described the open letter from 238 international aerosol scientists as “ *a little bit of a tempest in a teapot […]”* and reiterated her confidence in the existing advice focused on large droplets and surface transmission (source C3, Table 1 ^45^). It was not until early January 2021 that the British Columbia Center for Disease Control (BCCDC) edited its guidelines to include the risk posed by “smaller droplets” (which may be a euphemism for aerosols):

“ *Smaller droplets come out of the mouth and nose at the same time as larger droplets. These smaller droplets are light, and they can float in the air for a longer time. Because of this, smaller droplets may collect in enclosed spaces unless they are diluted with clean air from the outdoors or from a ventilation system. If many people are sharing a space without enough clean air, it can lead to COVID-19 infections.”* (source C6, Table 1 ^46^)

Despite stopping short of the use of words such as ‘aerosols’ or ‘airborne’, the new guideline was welcomed by front-line workers. But it still provided ambiguous messages on masks and omitted any directives to improve ventilation in schools. Extraordinarily, school officials in one locality ordered classroom windows to be screwed shut after teachers had opened them to increase ventilation (source C11, Table 1 ^47^).

The Provincial Health Officer for British Columbia is a highly-respected medical doctor. She had initial specialist training in infectious diseases and a distinguished career in military medicine, as well as working for the WHO internationally, and subsequently trained in public health. She was the public health lead in Toronto for the 2003 SARS outbreak, and in 2018 was the first woman to be named as chief medical officer for British Columbia. Her regional role during the Covid-19 pandemic, including regular media briefings to explain aspects of the science to the public, made her a household name in the state and conferred legitimacy and authority on her statements (source C12, Table 1). She appears at least partly driven by the urge to quell panic and maintain calm—which may explain her ‘storm in a teacup’ comment and, more generally, her reluctance to embrace theories about the dominance of aerosol transmission.

In Quebec, a dispute about airborne transmission of the virus ended up in the courts. In April 2020, during the first wave of the pandemic, Quebec’s Nurses Union (FIQ) asked the province’s National Institute of Public Health (INSPQ) that they mandate the use of N95 masks (a kind of respirator providing high-grade protection against aerosols) in long term care facilities where most of the COVID-19 cases were occurring (source C13, Table 1). However, INPSQ’s advice was based on a theory of large droplet and fomite transmission (source C16, Table 1) and this view shaped the institutional response. It was illustrated, for example, by a report from around the same time in which one regional hospital network publicly blamed employees’ sloppy handwashing to explain a COVID-19 outbreak (source C14, Table 1).

The first wave of the Covid19 pandemic took a very hard toll on Quebec’s long-term care residents and workers. Between March 2020 and February 2021, 13% of the 40,000 people institutionalized in a long-term care institution in Quebec died from Covid-19 (source C22, Table 1). In some institutions, virtually all residents became infected and so few workers remained that at one point the Canadian military was called in to take over (source C15, Table 1). Notably, the army provided N95 masks for all their troops undertaking this work (source C15, Table 1).

Nevertheless, aligning with the orthodox view, Quebec's Director of Public Health published an ordonnance on 8 ^th^ June 2020 forbidding the use of N95 masks for health professionals save for a few designated procedures (source C17, Table 1). On 10 ^th^ July 2020, after losing hope of finding a negotiated settlement on their request for N95 masks, the FIQ brought the matter to a labour tribunal (source C18, Table 1). The Nurses Union claimed there was no shortage of N95 masks and that denying nurses this higher-grade protection was going to cost lives (source C19, Table 1). The availability of sufficient N95 masks was later confirmed by the minister of health (source C20, Table 1). By that time, more than 17,000 health care workers had been infected (source C20, Table 1).

The legal action ended with a scathing legal ruling in March 2021. The employer had argued, through an expert witness from the Public Health Institute of Quebec (INSPQ), that there was no evidence of significant airborne transmission of SARS-CoV-2 and that standard medical masks were adequate protection. In upholding the nurses’ case, the judge decried INSPQ’s expert for using ‘false arguments’ and *‘*fallacies *’*, and another expert for being unaware of key scientific developments in his field when testifying *.* The ruling also noted that, *"despite [an] emerging scientific consensus, the INSPQ maintains, in court, the position that there is no air transmission"* (source C22, Table 1 paragraph 143). Interestingly, shortly afterward, a group of 109 health professionals, most of them doctors and nurses from infectious diseases backgrounds, wrote a public letter contesting the scientific soundness of the ruling (source C23, Table 1). The letter states, for example:

*“Given the lack of scientific evidence regarding the benefits of wearing a respirator over a procedural mask when in contact with ‘medium-risk’ patients, we suspect that your recommendations are likely heavily influenced by the ‘precautionary principle’. However, the precautionary principle must be based on science and not obscure it. In addition, this precautionary principle could prove to be deleterious for all workers in Quebec if the risks associated with your recommendations are greater than the expected benefits. We therefore ask that the CNESST [Commission des Normes, de l'Equité, de la Santé et de la Sécurité du Travail (French: Committee on Standards, Equity, Health and Safety at Work; Canada)]*
*carry out as soon as possible and publish a risk-benefit analysis of its recommendations. This analysis should include a forecast of the expected benefits including a quantification of the cases of infection prevented through these recommendations, as well as the number of RPAs that should be used to prevent infection in a worker (known as “Number needed to treat”). In addition, this analysis must include an estimate of the occupational injuries that will occur as a result of these recommendations. Prolonged wearing of the respirator is indeed associated with risks for workers (skin lesions, headaches, fatigue and increased risk of becoming infected, etc.).”* (source C23, Table 1)

The phrasing of the letter strongly emphasises the potential harms of the N95 mask (including, allegedly, a worker becoming so fatigued that they become inadvertently infected) rather than its potential benefits (preventing infection and death). The extract also illustrates the authors’ appeal to the tools of evidence-based medicine (such as the Number Needed to Treat metric ^48^) and their irritation in the face of what they appear to perceive as a challenge to their legitimate scientific authority in the sub-field of infectious diseases.

***Japan.*** In Japan, cases and deaths from Covid-19 were very low compared to the West but high compared to neighbouring countries like Taiwan ^49^. The first documented case of Covid-19 in Japan was on 15 ^th^ January 2020. By mid-February, there was an active national containment strategy in place which included assiduous contact-tracing with the goal of identifying, investigating, and quashing new clusters promptly ^50^. Contact-tracing was both prospective (to identify onward transmission) and – unusually – retrospective (to identify which past activities may have led to the person becoming infected). Japan’s approach also included lengthy quarantine periods (30 days), strict border controls, voluntary restriction of activities (for example, reducing travel and eating out, and working from home if possible), masking in the workplace, and economic support for businesses.

In contrast with the confident announcements from WHO and Western governments that the virus was not airborne, the Japanese government did not make any firm statements about the mode of transmission in these early documents. Notably, however, on 9 ^th^ March 2020, the Government of Japan (source D1, Table 1 ^51^), with the personal backing of the Prime Minister, reported a careful analysis of several clusters across the country made possible through meticulous analysis of retrospective contact-tracing data. It introduced the ‘3Cs’ message (avoid closed spaces, crowded places, and close proximity), explicitly invoking the precautionary principle on the grounds that the virus could be airborne:

*6. What we ask of you*
*The locations where mass infections were confirmed so far are places where the following three conditions were met simultaneously: (1) closed space with poor ventilation, (2) crowded with many people and (3) conversations and vocalization in close proximity (within arm's reach of one another). It is believed that more people were infected in such places. Therefore, we ask that you predict locations and settings where these three conditions could occur simultaneously and avoid them*.
*We do not have enough scientific evidence yet on how significantly such actions can reduce the risk of spreading infection. However, since places with poor ventilation and crowded places are increasing infections, we ask that you take precautions even before scientific evidence for clear standards is found.”* (source D1, Table 1 ^51^, page 2)

Central to Japan’s novel and successful strategy of retrospective contact tracing was a large cohort of highly-trained public health officers based in (or rapidly redeployed to) local public health centres – an approach sometimes referred to as field epidemiology. Importantly, these officers had ongoing experience of dealing with other airborne infectious diseases in the community, notably tuberculosis (TB), for which aggressive retrospective contact tracing with a view to identifying and controlling clusters was already in place ^50^.

Japan’s 3Cs message, which was designed to inform both national policy and public behaviour, was adopted into public-facing WHO advice on 13 ^th^ October 2020 (source A13, Table 1 ^52^), though the mismatch between this advice and continuing statements elsewhere there was “no firm evidence” for airborne spread ^32^ was not addressed. Retrospective contact tracing had been attempted in some Western settings (e.g., British Columbia) in the first wave of the pandemic but was not sustained or showcased in the same way as Japan, nor did analysis of case clusters persuade local public health officials of the airborne nature of the disease. 

The striking difference in how aerosol theory was received in Japan compared to our other case studies may be explained in part by an analysis of individual-level factors. For example, the personality and trajectory of the virologist who is credited with introducing Japan’s ‘3Cs’ approach to the pandemic back in March 2020 (source D6, Table 1 ^53^) - which structured his own habitus – likely played a role. He is a medical doctor by training and spent many years working as a senior adviser for the WHO on communicable diseases. He led WHO South East Asia’s response to SARS in 2003, where he became known for promoting transparency and information-sharing. But this doctor took a novel approach to the COVID-19 pandemic. He worked with a professor of mathematics to analyse early clusters of cases both within Japan and on the Diamond Princess cruise ship. The discovery that—unlike with influenza—a tiny proportion of primary cases gave rise to around 80% of secondary cases led them to hypothesise that the airborne route of transmission was dominant and that the key to controlling the pandemic was controlling clusters ^53^. A press article from November 2020 describes him thus:

*“X---, an unassuming and bespectacled 61-year-old, is at times hardly distinguishable from the average salaryman. A field epidemiologist by training, X--- cut his teeth working for Japan’s development agency in Zambia, and has spent most of his career as an academic, currently affiliated with Tohoku University. He’s far less well-known in his native country than other top infectious disease officials like Anthony Fauci in the U.S., and unlike Sweden’s Anders Tegnell, no one is tattooing his image on their bodies. But those who worked with X--- say his early sense of urgency, constantly badgering government officials to do more, was crucial to Japan’s response.”* (source D6 ^53^)

In contrast to many key public health actors in the West, then, this leading medical adviser in Japan kept a low public profile, decided that randomised controlled trial evidence and the assumptions of evidence-based medicine had little to contribute to the pressing questions around transmission, worked quickly and collaboratively with non-medical experts to produce a novel mathematically-informed hypothesis, embraced the precautionary principle (that action should be taken before scientific evidence is definitive), ignored advice from the WHO, developed a simple and catchy ‘3Cs’ message, and operated quietly but effectively behind the scenes to bring political actors up to and including the Prime Minister on board.

This contrasts with his contemporaries in the West, whose vested interests in their own game and their decision to reject the contribution of heterodox scientists tied them firmly to contingent paths. The Japanese adviser was able to enjoy interdisciplinary collaboration in a country which seems fairly open to interfield alignment and solidarity in relation to public health.

## Results

### Orthodoxy and heterodoxy

In this section we present the findings of a textual analysis of publications and statements from scientists relating to SARS-CoV-2 transmission. In sum, despite an appearance of a shared doxa, the scientific field contains deep divisions with distinct orthodox and heterodox positions. The orthodox position, taken by infectious disease researchers (mostly doctors working in hospital environments and schooled in the traditions and values of evidence-based medicine), can be summarised as follows. First, viral particles in exhaled air can be divided more or less straightforwardly into droplets (five microns or larger in diameter) or aerosols (below five microns); the former transmit the disease within two metres while the latter would account for any transmission (if it occurred) beyond two metres. Second, whilst the virus is clearly present in short-range respiratory droplets, lack of consistent identification of SARS-CoV-2 in air samples means there is no good evidence for the airborne theory of transmission. Third, the randomised controlled trial occupies a special position in the ‘evidence hierarchy’; other study designs, especially case studies, laboratory studies, and animal studies are considered inherently lower quality. Fourth, the technique of systematic review, in which a highly structured, methods-focused and somewhat technocratic approach is taken to searching, data extraction and data synthesis, is the best way to combine and summarise studies. Fifth, policies should be based on the findings of ‘high-quality’ research (i.e., systematic reviews, with an emphasis on methodologically ‘robust’ designs, especially randomised controlled trials) which directly addresses the policy question, not on theoretical speculation, ‘low-quality’ studies, or indirect evidence. Finally, systematic reviews of randomised controlled trials have demonstrated the benefits of handwashing, surface cleansing, and (in some circumstances) masking of healthcare staff and sick patients but not of masking asymptomatic members of the public, opening windows, or other kinds of ventilation, in respiratory disease prevention. In sum, this position upholds the droplet theory of SARS-CoV-2 transmission and supports prevention measures focused on handwashing, physical distancing (with two metres seen as a key cut-off level) and selective masking. It does not exclude the possibility of airborne transmission, but considers that further ‘high-quality’ studies are needed to strengthen the evidence base for policymaking ^6^. The orthodox position says little about the precautionary principle and does not actively embrace it.

The heterodox position, taken by aerosol researchers (mostly laboratory-based engineers and chemists who study the behaviour of particles suspended in the atmosphere), is as follows. First, aerosols are extremely complex; they vary considerably in size (up to 100 microns) and their flow follows turbulent, not linear, dynamics. Second, whilst aerosols account for all disease transmission beyond two metres, they transmit predominantly at close range (within one metre), hence close transmission cannot be attributed solely (or even predominantly) to droplets. Third, the evidence for aerosol transmission of SARS-CoV-2 is strong and comes from many different kinds of study, notably detailed analysis of ‘super-spreader’ events where dozens of individuals became infected from one or a small number of index cases, as well as laboratory studies including animal experiments. Fourth, policies can and should be based on a rich narrative synthesis of heterogeneous evidence, not solely (or even at all) on randomized controlled trials. Finally, given the dire implications of misplaced reassurance, a precautionary approach (changing policy when evidence suggests aerosol transmission, even if it falls short of scientific proof) may be justified. In sum, this position upholds a predominantly airborne mode of transmission and supports prevention measures focused on public masking and reducing ‘shared air’ (by avoiding crowding and increasing ventilation), in addition to handwashing and surface cleansing.

There is an uneven relationship of power and status between these orthodox and heterodox positions. The orthodoxy described above is considered and upheld as the prototypical science mainly within the field of medicine and to the extent that medical experts operate in a uni-disciplinary way and eschew cross-disciplinary collaborations—see for example the WHO’s Scientific Brief on Transmission of SARS-CoV-2, (source A11, Table 1 ^32^ and outputs published as highly-regarded Cochrane Reviews ^54, 55^. The heterodox perspective is considered legitimate among scientists more generally outside the field of medicine. The leading science journal, *Nature*, for example, has published numerous papers demonstrating airborne transmission of the virus – see for example some landmark animal studies ^56, 57^, an overview of superspreading events ^58^, and several editorials arguing for a paradigm shift from droplet and fomite to aerosol transmission ^31, 59, 60^. Another leading scientific journal, *Science*, has published empirical studies of transmission dynamics which support the airborne hypothesis ^61, 62^, a highly-cited short commentary ^4^, and a comprehensive narrative review ^63^. Occasional cross-fertilisation in academic journals is illustrated, for example, by the work of Bourouiba, an aerosol scientist whose papers generally appear in journals such as *Annual Review of Fluid Mechanics* ^64^ but who has successfully published in leading medical journals such as *JAMA* ^65^. Within the medical sub-field, clinical authors occasionally align with the heterodoxy – for example, in the journal *Anaesthesia* where there is much interest in the question of whether AGMPs are the only situations where higher-grade protection is needed ^66^.

Importantly, however, the heterodoxy remains largely an outsider within the hegemonic structures of medical science, and, in that context, it requires active recognition and support if it is to serve as a source of innovation from the margins. Scientists can publish research supporting the orthodox and heterodox positions, but academic debate appears to be largely a double monologue with both sides strengthening their own position but failing to convince—or even attempt to convince—the other sub-field. Public heath administrations, the de facto implementation channel for scientific findings in this context, have the power to arbitrate the scientific divide and select a preferred version of the truth. As Weiss showed, evidence use by policymakers is mostly strategic—that is, used selectively to justify a chosen position rather than dispassionately to inform it 67. As part of the State apparatus, public health administrations can use the State monopoly of symbolic violence to push what they uphold as truth (for example, in the media, in institutional guidance, and in lockdown policies). The premature decision of key public health bodies to favour a droplet over aerosol mode of transmission instead of letting scientific debates run their course had far-reaching negative consequences.

### Scientific capital and illusio

The two scientific sub-fields on which we focus have differently valued forms of scientific capital. Broadly speaking, in the West the scientific capitals of infectious disease scientists are symbolically valued and promoted as robust and relevant evidence, whereas the scientific capitals of aerosol scientists, who occupy a heterodox position, are framed as weak, soft, or limited. These framings present uneven power relations. Our data also powerfully illustrate the role of *illusio* (ie., the belief of players that the game they play is worthy of playing) in the unfolding of national and international infection control policy ^68^. *Illusio* is the allure of a game, which on the one hand draws in and keeps the players who find it worthy to be in a game, and on the other strips them of developing a healthy and critical view of the processes and outcomes of the game. By joining its routines and activities, individuals start accepting the rules of the game in a particular field as natural and objective, even when the game that they play may be to their own detriment or harmful to others. An epistemic break from the *illusio* is hard to achieve from within the game because the rules of the game appear innocuous to the players, who either join the game knowing them or come to accept them over time.

In the case of WHO, and those of the UK and Canada, the scientific capital of infectious disease scientists was recognised and valued above and beyond that of aerosol scientists. On the basis of the dataset we analysed, the former appears to have a particularly strong belief in the worthiness of their game. For example, a press article on the controversy around airborne spread in July 2020 (source A9) quotes a member of the WHO IPCRDEG-C19:

*“It is a shame that they [the 238 scientists who wrote the open letter] felt the need to publish,’ X---, a professor at the University of East Anglia in the United Kingdom and a member of WHO's infection prevention committee, told Live Science. ‘Given the ample evidence that reducing droplet transmission works [to reduce COVID-19 spread], throwing other things into the mix only confuses people and undermines the World Health Organization at a critical time,’ X--- said. […] ‘Most of them are chemists, engineers, owners of ventilation companies,’ X--- said. ‘They do not have a broad understanding of disease transmission mechanisms … this issue is more nuanced than many of them realize.”* (source A9, Table 1 ^30^)

Another article, published in *Nature* at around the same time (source A10, Table 1), quotes the lead for the WHO Secretariat for the IPCRDEG-C19:

*“There is this movement, which made their voice very loud by publishing various position papers or opinion papers,’ [Y---] says. ‘Why don’t we ask ourselves … why are these theories coming mainly from engineers, aerobiologists, and so on, whereas the majority of the clinical, infectious-disease, epidemiology, public-health, and infection-prevention and control people do not think exactly the same?”* ^31^

In both the above extracts, members of the IPCRDEG-C19 illustrate a number of elements of *illusio*: a shared sense of the rules of the game, depiction of the orthodox position as well-established truth arrived at through rigorous, ‘proper’ medical science (and as the benchmark against which all other science is measured), rejection of the heterodox position as flawed and based on weak science, and the view that adherents to the heterodoxy lack understanding of the topic, ignore well-established facts, and sow confusion by casting doubt on the orthodoxy. Indeed, the *illusio* appears to underpin the symbolic violence of the message that adherents to the heterodoxy are not entitled to voice their views because this threatens the stability of the orthodoxy. These excerpts also suggest a closing of rank among committee members, possibly reflecting features of the medical sub-field which—arguably—hosts a habitus of elite and exclusive rites of passage and dispositions among its members.

In a more recent example from our dataset, in a high-profile panel discussion broadcast live from a Canadian university in April 2021 (source A21, Table 1), the Chair of the WHO IPCRDEG-C19 warns his audience to “be careful” when evaluating the large amount of weak, non-peer-reviewed science on the topic of SARS-CoV-2 transmission (timed at 28.00 in the recording); he cites a flawed modelling study purporting to demonstrate airborne transmission that was later withdrawn (28:52). He reminds his audience that science needs to follow “basic epidemiological principles” (29:48) and that “this is fundamental infectious diseases epidemiology” (29:55) (emphasis added). Later in his talk (40:13), he challenges aerosol scientists’ claim that the virus is difficult to culture from the air (he describes it as a *"[r]elatively easy virus to culture and is everywhere in the environment"*) and emphasises how his own team have cultured the virus repeatedly from droplets. There are, he states “very few studies of high quality” published by aerosol scientists (45:10). While acknowledging the likelihood of what he calls “situational” airborne transmission (by which he appears to mean rare spread when an unusual combination of environmental conditions are found), his conclusion is *“I would like to see a much higher level of scientific evidence, including some basic science”* (56:10) before policy changes.

Thus, the chair of the WHO committee charged with writing international guidance on SARS-CoV-2 transmission anchors his arguments firmly in the orthodoxy of his own medical sub-field. He depicts the relatively sparse and inconsistent evidence base from air sampling studies of SARS-CoV-2 as due to poor scientific technique and ignoring “basic” scientific principles. Crucially for someone in a position of international policy influence, he sees no need for policy to change.

Symbolic violence—the exertion of power by the orthodoxy to impose its own norms on less powerful groups—is on display in the confident statements made by its adherents (see also C23, Table 1). But the practices and discourses of symbolic devaluation are also produced and reproduced in a more subtle way by those whose work is symbolically violated, as they—to a greater or lesser extent—accept or acquiesce to their lowly stakes in the game. Notwithstanding the important example of the open letter to policymakers from aerosol scientists (source A8, Table 1 ^3^), protests from aerosol scientists during the pandemic have been relatively few. It would appear that at least some aerosol scientists have remained silent, accepting the way their evidence is relegated to secondary status through subtle and overt mechanisms of devaluation.

Our Japanese case provides an interesting contrast. From the outset, Japan accepted an airborne mode of transmission and its government acknowledged and highlighted research by aerosol scientists at leading Japanese universities ^69^, allowing their scientific capital to be recognised. Of course, aerosol scientists existed in the West, and indeed outnumbered those from the East in the open letter to policymakers and aimed at the WHO ^3^. What appears to be missing from the Japanese case is a powerful counter-lobby of infectious disease scientists, aligned strongly with droplet theory and the evidence-based medicine movement, whose modus operandi was to focus predominantly on the findings of randomised controlled trials and warn policymakers against taking action until such so-called ‘high-quality’ evidence was available. This case, and parallel cases elsewhere in the far East, may partly reflect historical differences such as the relatively recent experiences with SARS (2001) and MERS (2012). But it may also reflect a greater openness within the scientific field to multiple disciplines and sub-fields bringing their expertise together to address a crisis. This in turn may be because, for various reasons, the sub-field of evidence-based medicine with its formal hierarchies of evidence and its close links to top-down, standardised approaches to health policy ^70, 71^ never became orthodoxy in Japan ^72^. 

It is also worth noting how, in Japan, teasing out the detail of local clusters of COVID-19 led to abductive reasoning of the format “what could have caused this?” and immediate mobilisation of the precautionary principle for a national 3Cs policy before the science of airborne transmission was confirmed. This contrasts markedly with the more superficial approach to cluster analysis taken by most Western countries which appeared to presuppose a droplet mode of transmission. The US Centers for Disease Control and Prevention, for example, published a weekly report in May 2020 describing a large outbreak of Covid-19 following a choir practice and commenting – prematurely – that *“choir practice attendees had multiple opportunities for droplet transmission from close contact or fomite transmission”* ^73^. The outbreak was later shown, through meticulous analysis of interpersonal interactions and who exactly touched which shared objects such as chairs and food plates, to be due to inhalation of airborne virus ^74^.

Despite Japan’s markedly different approach in which aerosol science was considered legitimate evidence for informing the pandemic response from as early as February 2020, its ‘3Cs’ message was adopted only belatedly by the WHO and given limited emphasis (source A13, Table 1). This begs the question: when scientific orthodoxy differs across countries and regions, whose orthodoxy will gain a hegemonic hold in the international public health space, and why?

### Intersecting fields

Our case study of the droplet (orthodoxy) versus aerosol (heterodoxy) stand-off, exemplified at international level by the WHO’s stalling and at national level by various local, regional, and national conflicts over worker protection, illustrates the intersection of the three key fields – political, state (policy and regulatory) and scientific – introduced in our theoretical section above.

Politically, when the droplet theory was embraced in the WHO Director-General’s emphatic self-correction ^75^, it distanced the WHO from the “military word” (that is, linked to bioterrorism and hence with major political overtones and implications far beyond health) and brought the new disease into a less overtly political and more narrowly health-focused semantic space. It also provided an apparently robust scientific rationale for the potentially politically sensitive decision to provide only lower-grade protection for most healthcare workers at a time when global supply chains for higher-grade PPE were perilously inadequate ^26^.

Alignment between the WHO’s espoused scientific position and the State (bureaucratic public health) field in different countries was achieved largely through the production of position statements, scientific briefings, and guidelines, underpinned by systematic reviews of evidence, often in the form of what is known as ‘living’ reviews (that is, periodically updated to incorporate new evidence)—though as our empirical data illustrate, such documents rarely employ new thinking. How such guidance documents were produced is described in the following quote from a WHO press conference on 5 ^th^ June 2020, in which the lead for the WHO’s Secretariat responds to a journalist’s question:

*“our process of developing guidance is based on … all the existing evidence and then through a process of consultation of international experts from different countries and different disciplines. Of course this topic is dealt with mainly by infection prevention and control, infectious diseases and epidemiology specialists. Many of these people actually are health workers who take care of COVID-19 patients so we consulted these experts and evaluated a variety of evidence; first of all the evidence about the modes of transmission of this virus, which so far have been demonstrated for droplet and contact.[..]*
*The second element of the evidence is the evaluation of the effectiveness of the face protections and there are randomised control trials which are the best type of evidence we can wish that demonstrate no difference in effectiveness in preventing transmission of influenza or other respiratory viruses. Eventually there is also some emerging evidence from observational studies which unfortunately have a lower level of evidence […].”* (source A7, Table 1, emphasis added ^76^)

In this response, the WHO representative firmly locates relevant expertise in the medical orthodoxy of infectious disease science. Indeed, it was because the experts were directly involved in patient care (i.e., were doctors) that their expertise was privileged in the production of WHO advice. In stating that “ *all the existing evidence*” ^74^ had been considered and uncritically endorsing evidence-based medicine’s methodological hierarchy with its privileging of randomised controlled trials (see also sources A20 and C23, Table 1), she appears to designate the work of aerosol scientists as either not evidence at all or as inherently poor-quality. Having dismissed this heterodox evidence, and drawing only on medical orthodoxy, she concludes that the evidence base for droplet transmission is well-established whereas that for aerosol transmission (beyond AGMPs) is weak or absent. Thus the state field aligns strongly with medical scientific orthodoxy and systematically excludes heterodoxy while appearing to include “ *a variety of evidence*” ^74^.

A similar orthodoxy-driven alignment between political, state, and scientific fields is evident in our national case studies from the UK, Canada, and USA. In the UK case, for example, the WHO’s early—incorrect—statements emphasising droplet infection were set in stone in very first draft of an influential national guideline; subsequent updates just added detail (and hundreds of references) but did not question the fundamental model of transmission or the precautions based on them ^42^. The aerosol scientists’ heterodoxy had so little credibility that their protests to the WHO could be described using trivialising language as *“a tempest in a teacup”* by local public health officials in Canada (source C3, Table 1 ^45^). Japan’s strong alignment with aerosol heterodoxy, including its early mobilisation of the precautionary principle (source D1), from the outset provides striking contrast here.

The push-back against medical scientific orthodoxy in the West came from relatively low-status staff such as non-medical healthcare workers, classroom teachers, and shop-floor workers whose training encourages them to provide a quality, hands-on service to the patient, pupil, or customer. These workers had begun to suspect that they were at risk of an occupational disease because they had seen colleagues succumb, but who had little individual control over their working conditions or environment. The response of the state was overtly political – for example in the individualization and financialization of responsibility and advice to workers to “use their professional judgement” (using the *illusio* of professional choice) and purchase their own higher-grade PPE if they felt it was needed since there was ‘no evidence to support a change [in policy]’. The involvement of workers’ unions and resort to the courts to push the responsibility back on employers brings in the legal sub-field, whose dismissal of droplet orthodoxy in one case is swift and emphatic, highlighting how medical orthodoxy does not always survive the test of rigour in other fields.

Symbolic violence also emerged at the interplay of social and state policy subfield in the shape of various infection control rituals introduced in the early months of the pandemic. Whilst the introduction of hand sanitisers, masking and physical distancing measures in public places was a plausible response (all were probably based on a droplet theory of transmission, though the latter two would also reduce aerosol transmission), other measures seemed heavy-handed and misaligned with evidence. Closure of parks, beaches, open-space exercise areas, and children’s playgrounds as possible hotspots of infection, for example, occurred in both Canada and the UK. Such measures over-emphasise the risk of droplet infection (e.g., through contaminated equipment) and ignore the vastly reduced risk of transmission outdoors (since aerosols are quickly dispersed) compared to indoors ^77^; they appear to represent a highly symbolic move in which safe and familiar local places and spaces traditionally associated with health and refreshment come to be redefined by the state, who claim to be ‘following the science’, as dirty and unsafe. In the same way, screwing classroom windows shut in schools in our Canadian case study (source C11, Table 1) is an example of symbolic violence manifested in hegemonic inter-field struggles (since such a measure does not conflict with a droplet theory of transmission but is dramatically misaligned with aerosol transmission).

The dominant discourse of medical scientific orthodoxy, once it finds allies in economic ideology and political concerns such as limited resources for preventative measures and PPE could potentially become an instrument of symbolic violence to taint, trivialise, denigrate, and ultimately control professional and individual choice in other fields (for example, in the private sector where employers claim they are following evidence-based hygiene practices by installing hand sanitisers but refuse to impose mask mandates or invest in expensive ventilation or air filtration measures to protect employees and customers ^78^).

### Emerging challenges to orthodoxy

Whilst the message from aerosol scientists that transmission of SARS-CoV-2 is predominantly airborne was ignored and dismissed within the medical and health policy mainstream during 2020, the heterodoxy did not die. On the contrary, by spring 2021 the dominance of airborne transmission was becoming more accepted even within medical circles – for example, in January 2021 the *Journal of Hospital Infection* published a paper entitled ‘Dismantling myths on the airborne transmission of severe acute respiratory syndrome coronavirus-2 (SARS-CoV-2) *’* ^79^, and in April 2021, the *British Medical Journal* commissioned an editorial from an overlapping team entitled ‘Covid-19 has redefined airborne transmission’ ^75^, the *Lancet* published ‘Ten scientific reasons in support of airborne transmission of SARS-CoV-2’ ^2^ and the *Journal of the American Medical Association* published a review article on ventilation and filtration ^80^. Whilst the WHO remained resistant to the phrase ‘airborne transmission’, it placed increasing emphasis on ventilation in late 2020 and into 2021 (source A17, Table 1 ^38^).

It is worth examining the authorship and presentation style of papers supportive of aerosol spread which were published in mainstream medical journals. For example, Professor Tang, the lead author on two papers cited in the previous paragraph ^75, 79^, is a medical doctor (virologist) but has a track record of working on interdisciplinary studies with laboratory scientists. He was already working on airborne transmission of respiratory infections, as illustrated by a 2019 paper ^81^ on chickenpox, measles, tuberculosis, influenza, and smallpox– all diseases which have been shown to be airborne (some after a long delay). Another doctor on the ‘Dismantling myths’ paper, Raymond Tellier, gained an undergraduate degree in mathematics and a MSc in physics before going on to become a professor of infectious diseases.

Rather than simply setting out the aerosol science arguments, Tang and his co-authors engage in an effort to persuade. They construct their review paper around what they depict rhetorically as “myths” – namely, elements of the medical orthodoxy described above ^5^. They begin by acknowledging the assumptions of the orthodox scientists, but then explain why those assumptions are flawed. In their editorial, in relation to a key terminological confusion around words like ‘droplet nuclei’ (a term used by droplet scientists to depict exhaled droplets which have evaporated down to five microns or smaller, are suspended in the air, may persist, and travel long distances, but still—in their terminology—merit the name ‘droplet’), they state:

*“The confusion has emanated from traditional terminology introduced during the last century. This created poorly defined divisions between “droplet,” “airborne,” and “droplet nuclei” transmission, leading to misunderstandings over the physical behaviour of these particles. Essentially, if you can inhale particles—regardless of their size or name—you are breathing in aerosols. Although this can happen at long range, it is more likely when close to someone, as the aerosols between two people are much more concentrated at short range, rather like being close to someone who is smoking.”* (page 913) ^79^

In sum, papers written by interdisciplinary groups and including medically-qualified individuals with experience in other sub-fields have begun to systematically challenge the orthodoxy from within its own sub-field. Such publications, however, remain sparse at the time of writing.

## Discussion

In this paper, we have used the detailed analysis of local, national, and international case studies to show how, through repetition of a hierarchically-constructed scientific game, a hegemonic order emerged between the orthodox position taken by clinically-qualified infectious disease scientists in the West who aligned with the evidence-based medicine movement and supported a predominantly droplet mode of transmission for SARS-CoV-2, and the heterodox position taken by aerosol scientists supporting a predominantly airborne mode, to the exclusion and dismissal of the latter group and their evidence. The *illusio* in this case was upheld with the hegemonic structures of state regulators who held on to a limited range of evidence that called for less costly measures, and international agencies such as WHO, whose committees and decision-making groups on this particular topic showed a high degree of internal agreement and lacked interdisciplinarity in their expertise and methodologies. The proximity (in training and organizational position) between medical science and State public health ensured that the de facto arbiters of truth strongly favoured the orthodox narrative.

We have shown how the nature of the scientific game benefited the orthodox scientists, enhanced their status and depicted the heterodox scientists’ claims as vocal but incorrect or irrelevant. However, the mounting evidence in support of a dominant airborne route of transmission has begun to produce a gradual shift, whereby the *illusio* that the orthodoxy created is increasingly contested. Through consistent claims of legitimacy and recognition, aerosol science has managed to encroach on the orthodox position internationally. But this has happened only to a limited degree and at a pace that is quite unsuited to an urgent pandemic response. The partial and gradual nature of the change, and the persistence around the world of policies underpinned by flawed droplet science, begs the question of what could be garnered as lessons from this experience. We posit that there is need to improve the mechanisms by which multiple disciplines gain legitimate access to be considered as evidence by regulatory and advisory organisations, and also to improve the measures of accountability by which such organisations judge themselves and are judged by others.

According to Bourdieu, fields and subfields have their own ontology, their particular ways of knowing and affective engagement with knowledge ^82^. Bourdieu explained how such inter-field relations operated between fields of science and society: *"Strictly scientific authority tends to convert itself, over time, into a social authority capable of opposing the assertion of a new scientific authority. Further, social authority within the scientific field tends to become legitimized by presenting itself as pure technical reason, and also the recognized signs of statutory authority modify the social perception of strictly technical abilities"* ^14^, page 7). Nowhere is this principle more evident than in the appropriation of terms such as ‘rigorous’, ‘robust’, and ‘evidence-based’ by the medical orthodoxy to define its own assumptions and interpretations as unassailable.

In order for fields and subfields to respect, recognise, and value knowledge in other fields or subfields they need to have ontologies that are pluralist. Such pluralism is often advocated under the banner of transdisciplinarity and interdisciplinarity, which are widely advocated in science but often fail to occur. Indeed, in relation to the living guideline on COVID-19, the World Health Organisation presents its *’*Guideline Development Group (GDG) comprising individuals with broad expertise spanning multiple specialties’ (source A16, Table 1 ^39^), its description of the guideline development process for COVID-19 is firmly locked to epidemiological assumptions (e.g., it recommends that research questions be framed as “PICO”—population-intervention-comparison-outcome—and uses a hierarchy of evidence in which randomised controlled trials sit above laboratory and case studies) ^83^.

Dominance of this exceptionally narrow ontology both within the WHO and also at the level of state regulators led to siloed and purist approaches by which the heterodox position (based on quite different kinds of research question and study designs) was trivialised, undermined, and systematically disregarded. Bourdieu hinted at an explanation for this uneven relationship: *"[t]he dominant agents are those who have the power to impose that definition of science according to which the most accomplished science consists of having, being, and doing what they themselves have, are, and do"* (page 14 ^14^). Whilst we acknowledge that practical issues—notably the global shortage of personal protective equipment mentioned above ^26^—also had a role to play, such influences did not explain why positions became even more entrenched when these shortages subsided.

The arbitration processes between state regulators and the scientific community that led to firmly-held, state-sanctioned positions on matters such as modes of transmission of SARS-CoV-2 or effective preventative measures cannot be understood if we conceptualize them as taking place entirely within the field of science. Whilst symbolic struggles within science can be understood on one level as Kuhnian paradigm struggles in which old theories and methods are increasingly unable to explain new observations and are therefore replaced ^84^, there is also a more political dimension about which Kuhn had little to say but which Bourdieu viewed as central. Dominant agents in the scientific field (doctors and epidemiologists) may have continued to defend the old paradigm long after evidence emerged to challenge it because their own power and status was linked to their involvement in the orthodoxy. Emerging agents from heterodox fields (chemists, ventilation flow modelers and engineers) found it difficult to challenge orthodox position on the legitimacy of methods and disciplinary contributions because they lacked power.

We believe that the COVID-19 crisis profoundly realigned the interdependence and relative autonomy of the three fields at the core of the response. Highly technical scientific debates regarding the transmissibility of coronaviruses—debates about which nobody but a handful of specialists would have had an interest in normal times—quickly became the point of intersection of science, politics, and policy. Dominant agents from the political field held daily press conferences to present and defend national COVID-19 prevention strategies and public health orders that directly influenced the daily lives of billions—and which in turn hinged on underlying theories about the assumed mode of transmission of SARS-CoV-2. In this context, the arbitration between competing *doxas* could not be left to its fate within the field of science. It became a matter of (political) life and death to agents in the political field as well as a central issue structuring political games, and the very *raison d'être* of organisations in the subfield of public health.

During 2020, political agents and public health structures in the West who were forced to make tough decisions invariably opted for the questionable orthodoxy of droplet-based transmission. We question whether the reluctance from those agents to consider airborne transmission—or even to take the precautionary principle into account—may be too systematic and too persistent to be explicable by the nature of the scientific evidence alone. A reviewer of a previous draft of this paper raised an important point:

*“Although infection control specialists may have been misinformed, they considered their views to be evidence-based. They did not reject heterodox views to defend the primacy of their field and defend their ‘scientific capital,’ but rather because they believed their knowledge to be superior and interpreted their observations in the light of an anachronistic paradigm.” [reviewer 1]*

To clarify, both Bourdieusian and a Kuhnian analyses assume, broadly speaking, that beliefs within a scientific orthodoxy are honestly held—precisely because they align with prevailing mental models. However, it is also the case that when a mental model is associated with a high degree of scientific capital (that is, when it is associated with power and prestige for the individual or group), the honest scientist will be less inclined to consider changing it. Such is the nature of illusio in many fields of science, the allure of the scientific game and the way the powerful players play it with great success over time prevents the players from developing a healthy view of the game once they are heavily vested and entrenched in it.

Our dataset contains several examples—such as the WHO’s reluctance to use the ‘military word’, and the declaration from both Scottish and Quebec officials that there was ‘no evidence’ to support a change to high-grade PPE for healthcare workers—consistent with subtle influence of political factors. In all these examples, refusal to adopt the precautionary principle was striking. Although the rationality of this political process could be questioned from a purely scientific perspective, it makes sense given the competing nature of priorities (financial, geopolitical, and practical).

Interdisciplinarity is an espoused practice in scholarship, yet, its operationalisation in terms of understanding reality often remains unattended ^85^. In this paper we suggest that embracing and protecting interdisciplinarity may be a key way to transcend fruitless struggles for power among subfields of science, politics, and medical practice. Ironically, interdisciplinarity is differently defined by different disciplines. More positivistic disciplines tend to view it in terms of collaboration—the combining of particular skills and knowledge bases to address a large and complex research challenge (as in computational genomics, for example). Following Rowland ^86^, we prefer to define interdisciplinarity as contestation—in which the meetings between disciplines lead to fundamental clashes (for example about the nature of reality or about preferred ways of knowing that reality) and inevitable conflicts. This latter kind of interdisciplinarity could either have virtuous (e.g., generation of new knowledge and insights) or vicious (e.g., pursuit of status and privilege) outcomes. In order to generate virtuous outcomes from interdisciplinary contestation, a better process of scientific governance is needed in key decision-making bodies as well as in design and delivery of research works.

Albert *et al*. ^87^ have suggested that in the context of academia, inclusive work practices could help interdisciplinarity to flourish between social sciences, humanities, and medicine. We believe that, in addition, radical changes are needed to governance in the key advisory bodies within which scientific topics are scoped, advice is commissioned, and findings assimilated and actioned. Rejection or under-emphasis of the airborne hypothesis for so long by bodies whose advice impacted on the lives of billions was, we believe, not merely a failure of the scientific process but a failure of the governance of that process. Effective governance requires not merely structures, processes, and technical procedures (which, as currently set up, assume that science sits separately from politics) but measures which recognise that science is politically entangled and therefore provide opportunities for actors to deliberate collectively and harness their differences and conflicts productively ^88, 89^. Rather than assigning power to a ‘closed shop’ of experts in a single scientific sub-field, thereby aligning with what Nowotny *et al.* ^90^ have called ‘Mode 1 knowledge translation’—defined as hegemonic, hierarchical, unidisciplinary and with a unidirectional flow of knowledge from science to policy—we exhort the WHO and other public health bodies to embrace what Nowotny *et al.* call ‘Mode 2 knowledge production’—an approach to science which is more socially distributed, more application-oriented, inherently trans-disciplinary, and subject to multiple accountabilities. In this second mode, a much wider range of stakeholders, including not just other scientists but also front-line workers and the lay public, would be involved in defining the questions for which different kinds of science might begin to provide answers. How such a model would play out in practice is the subject for another paper.

## Conclusion

In conclusion, the hegemonic grip of prevailing infection control discourse remains strong. We believe that exit from the pandemic depends on science and policy finding a way to renegotiate what Bourdieu called the ‘rules of the scientific game’ – what counts as evidence, quality, and rigour in pluralist rather than purist ways.

**Table 1.** Sources used in the case studies.

Setting	Focus	Sources analysed for this case (listed in date order)
International policy context	Early and continuing alignment of WHO policy with droplet mode of transmission	A1. WHO guidance on biological and chemical weapons (2004) ^25^ A2. Transcript of WHO inaugural press conference on Covid-19 (11 ^th^ Feb 2020) ^75^ A3. WHO interim guidance on use of personal protective equipment by healthcare staff (27 ^th^ Feb 2020) ^91^ A4. WHO video for the lay public on how to protect yourself against Covid-19 (28 ^th^ Feb 2020) A5. WHO Tweet “Covid-19 is not airborne” (28 ^th^ Mar 2020) A6. Asian Development Bank report on global PPE supply (April 2020) ^26^ A7. Transcript of WHO press conference (5 ^th^ June 2020) ^76^ A8. Journal article ( open letter to policymakers on behalf of 238 aerosol scientists) ‘It is time to address airborne transmission of coronavirus disease 2019 (COVID-19)’ by Morawska and Milton (6 ^th^ July 2020) ^3^ A9. Press article by Krisch on source A8 reporting interviews with WHO senior staff and committee members ‘Is the coronavirus airborne?’ (7 ^th^ July 2020) ^30^ A10. News feature by Lewis in *Nature* ‘Mounting evidence suggests coronavirus is airborne’ (8 ^th^ July 2020) ^31^ A11. WHO Scientific Brief ‘Transmission of SARS-CoV-2’ (9 ^th^ July 2020) ^32^ A12. Peer-reviewed article ‘Use of medical face masks’ by Conly *et al*. (6 ^th^ August 2020) ^7^ A13. WHO public information site ‘Coronavirus disease: Advice for the public’ (13 ^th^ Oct 2020) ^52^ A14. Letter to editor critiquing source A10 ‘Scientific evidence supports aerosol transmission of SARS-CoV-2’ by MacIntyre and Ananda-Rajah (18 ^th^ Dec 2020) ^36^ A15. Blog by Professor Raina MacIntyre ‘The hijacking of public health’ (5 ^th^ Jan 2021) ^92^ A16. WHO living clinical guideline on Covid-19, with linked biographies of guideline authors (updated 25 ^th^ January 2021) ^39^ A17. WHO guidance ‘ Roadmap to improve and ensure good indoor ventilation in the context of COVID-19’ (5 ^th^ Mar 2021) ^38^ A18. Paper launching WHO’s ‘clean your hands’ campaign led by Prof Allegranzi *et al*. (17 ^th^ Mar 2021) ^29^ A19. Evidence summary (preprint) ‘SARS-CoV-2 and the role of airborne transmission [version 1]’ by Heneghan *et al*. (31 ^st^ March 2021) A20. WHO document ‘ Update on WHO COVID-19 guidelines development’ (7 ^th^ April 2021) A21. Video of panel discussion between aerosol scientist (Prather), infectious disease epidemiologist (Fisman) and chair of WHO IPC R&D working group (Conly) at University of Calgary, Canada (9 ^th^ April 2021) A22. Royal College of Nursing Infection Prevention and Control Network website (undated).
UK	Ventilation and staff protection in healthcare settings	B1. COVID-19 Infection Prevention and Control (IPC) guidance (UK) (updated 21 ^st^ Jan 2021) ^41^ B2. Antimicrobial Resistance and Healthcare Associated Infection (ARHAI) rapid review on COVID-19 (v 11, updated 5 ^th^ Feb 2021) ^42^ B3. Open letter to UK Prime Minister (led by Royal College of Nursing) on protecting healthcare workers (19 ^th^ Feb 2021) ^40^ B4. Independent evidence review of guidelines for infection prevention and control in UK hospitals by Gould and Purssell, commissioned by Royal College of Nursing (28 ^th^ Feb 2021) ^43^ B5. Radio interview Good Morning Scotland (2 ^nd^ Mar 2021) (transcript available from authors) B6. Public Health England guidance on ventilation of indoor spaces (5 ^th^ Mar 2021) ^44^ B7. Blog by Professor Alison Leary ‘Why does healthcare reject the precautionary principle?’ ^93^
Canada	Ventilation and masks in schools (British Colombia) and staff protection in healthcare settings (Quebec)	C1. Tweet from British Colombia Centre for Disease Control @CDCofBC (11 ^th^ Feb 2020) “this is not an airborne virus”. C2. News report by Kotyk ‘“Absolutely no evidence” that COVID is airborne’ (1 ^st^ June 2020) C3. News report by Lindsay ‘Controversy over airborne transmission of Covid-19’ (20 ^th^ July 2020) C4. British Colombia Teachers’ Federation news release ‘ BCTF files application with the Labour Relations Board over COVID-19 health and safety concerns’ (18 ^th^ September 2020) C5. British Colombia Teachers’ Federation. Brief for the Minister of Education, the Honourable Jennifer Whiteside. (17 ^th^ Dec 2020) ^94^ C6. British Colombia Centre for Disease Control: How it [COVID-19] spreads (5 ^th^ Jan 2021) ^46^ C7. British Colombia Nurses’ Union web report ‘ BCCDC Now Recognizes Airborne Transmission of COVID-19’ (8 ^th^ Jan 2021) C8. British Colombia Centre for Disease Control: ‘Personal Protective Equipment’ (27 ^th^ Jan 2021) ^95^ C9. British Colombia Centre for Disease Control: ‘ COVID-19 Public Health Guidance for K-12 Schools’ (4 ^th^ Feb 2021) ^10^ C10. British Colombia Teachers’ Federation news release ‘ Changes to K–12 COVID-19 guidelines include some important positive steps, but more can be done’ (4 ^th^ Feb 2021) C11. News report by Brend ‘ School officials order windows screwed shut’ (20 ^th^ Feb 2021) C12. News report by Migdal ‘ 4 Takeaways from Dr Bonnie Henry’s New Book’ (12 ^th^ March 2021). C13. News report by Radio-Canada ‘ Le personnel médical en CHSLD veut davantage de protection’ (20 ^th^ Apr 2020) C14. Lafrenière, M. Le CIUSSS et le lavage des mains: « extrêmement petit de la part de la direction». newspaper article in Le Nouvelliste. (2020, Mardi 28th April 2020). C15. News report by Radio Canada (14th May 2020) Tous les résidents du CHSLD Vigi Mont- Royal ont contracté le virus. C16. Paré, I. Hécatombe chez les travailleurs de la santé. newspaper article in Le Devoir. (15th May 2020). C17. Public Health Ordonance : Arruda, H. (8 ^th^ June 2020). *Ordonnance du directeur national* *de santé publique concernant le port des équipements de protection respiratoires et oculaires*. Québec: Ministère de la Santé et des Services sociaux du Québec. C18. News report by La Presse canadienne (10th July 2020). https://ici.radio-canada.ca/ nouvelle/1718984/restriction-masque-n95-covid-coronavirus-cour-contestation C19. Paré, I. newspaper article in Le Devoir. (13th July 2020). Les infirmières contestent en cour le recours «abusif» à l'arrêté dictant leurs conditions de travail C20. Paré, I. newspaper article in Le Devoir (16th October 2020). Québec n’aura plus besoin de rationner les masques N95. C21. News report by M. Meloche-Holubowski (16 ^th^ February 2021) https://ici.radio-canada. ca/nouvelle/1768418/10000-morts-deces-covid-quebec-pandemie-covid-coronavirus C22. Legal judgement from Quebec court adjudicating between nurses’ unions and Public Health Institute of Quebec. (23 ^rd^ Mar 2021) C23. News report by Radio Canada (15th April 2021) La CNESST invitée à revoir sa position sur le port du masque N95 en zone tiède https://ici.radio-canada.ca/nouvelle/1785276/ directives-obligation-apr-patients-cas-tiedes-covid-19
Japan	Early implementation of ‘3Cs’ public information campaign	D1. Report from national expert meeting of Novel Coronavirus Control Team (Office of Japanese Prime Minister, 9th March 2020) ^51^ D2. ‘3Cs’ policy information poster (28th March 2020) ^96^ D3. Speech by Japanese Prime Minister Shinzo Abe (7th April 2020) ^97^ D4. Covid-19 update from Government of Japan (7th April 2020) ^98^ D5. Academic paper on COVID-19 in Japan by Shimizu and Nigita (23rd Oct 2020) ^49^ D6. Press article by Du ‘ This virus expert saved Japan from the worst of COVID-19. But has the magic worn off?’ (20 ^th^ November 2020) ^53^ D7. Academic paper on Japan’s public health centers by Imamura *et al*. (18th Dec 2020) ^50^

## Data availability

All data underlying the results are available as part of the article in Table 1 and no additional source data are required.
