# Supplementary material for: Orthodoxy, illusio, and playing the scientific game: a Bourdieusian analysis of infection control science in the COVID-19 pandemic
Source: Wellcome Open Res. 2021 Oct 22;6:126. Originally published 2021 May 24. [Version 3] doi: 10.12688/wellcomeopenres.16855.3 (PMC8474098; doi:10.12688/wellcomeopenres.16855.3)
Supplement: Peer review history

# Peer review: Reviewer response for version 2

**DOI:** 10.21956/wellcomeopenres.18903.r45984

**Published:** 2021-10-18

**Authors:** Ferric Fang

**Affiliations:** 1 Department of Laboratory Medicine and Pathology, University of Washington, Seattle, WA, USA

The authors have responded constructively to my earlier review.

 Now that one of the national case studies has been deleted from the article (USA), the abstract should be modified accordingly to indicate *three *national ones (UK, Canada and Japan), and references to the USA study should be deleted from the abstract and text.

Is the work clearly and accurately presented and does it cite the current literature?

Yes

If applicable, is the statistical analysis and its interpretation appropriate?

Not applicable

Are all the source data underlying the results available to ensure full reproducibility?

Yes

Is the study design appropriate and is the work technically sound?

Yes

Are the conclusions drawn adequately supported by the results?

Yes

Are sufficient details of methods and analysis provided to allow replication by others?

Yes

Reviewer Expertise:

-

I confirm that I have read this submission and believe that I have an appropriate level of expertise to confirm that it is of an acceptable scientific standard.

# Response

**Published:** 2021-10-18

**Authors:** Trish Greenhalgh

**Affiliations:** Nuffield Department of Primary Care Health Sciences, University of Oxford, UK

This reviewer has spotted some typos. We will see how we can get them corrected! Many thanks.

# Peer review: Reviewer response for version 2

**DOI:** 10.21956/wellcomeopenres.18903.r45983

**Published:** 2021-09-23

**Authors:** Florence Villesèche

**Affiliations:** 1 Copenhagen Business School, Copenhagen, Denmark

The authors replied to the comments thoroughly, clearly indicating where they agreed and disagreed, and how this led them to clarify or modify the text. 

 I am thus satisfied with the changes and have no further comments to make.

Is the work clearly and accurately presented and does it cite the current literature?

Yes

If applicable, is the statistical analysis and its interpretation appropriate?

Not applicable

Are all the source data underlying the results available to ensure full reproducibility?

Partly

Is the study design appropriate and is the work technically sound?

Yes

Are the conclusions drawn adequately supported by the results?

Partly

Are sufficient details of methods and analysis provided to allow replication by others?

Yes

Reviewer Expertise:

Diversity, identity, networks, organisational sociology

I confirm that I have read this submission and believe that I have an appropriate level of expertise to confirm that it is of an acceptable scientific standard.

# Peer review: Reviewer response for version 1

**DOI:** 10.21956/wellcomeopenres.18592.r44048

**Published:** 2021-07-07

**Authors:** Florence Villesèche

**Affiliations:** 1 Copenhagen Business School, Copenhagen, Denmark

This article adopts a Bourdieusian framework to frame and analyse the ‘scientific game’ related to infection control science in the Covid-19 pandemic. Specifically, the authors examine two transmission modes that have received highly unequal consideration: droplet vs airborne transmission, with severe consequences for how infection control is realised in practice. I find the article very insightful and rich and hope that my comments can help the authors improve the rendition of their reasoning.

 Overall, it is evident that the authors care deeply for this discussion, which occasionally translates into the paper making conclusions before providing the evidence. Also, and related, there may be possible amendments to better distinguish between the theorisation and the study results while also better integrating the data and the theory.

*Introduction*

 The starting quote is excellent and gives us a good sense of where you want to go with the paper - year missing in the reference, though. I also like that you start the article with a description of the phenomenon, what is happening, what an official statement looks like, and how it features only one possible transmission theory. However, I think it is a bit unwarranted to straight away state that this statement is wrong. I understand that this is, ultimately, the point you want to make, but you are jumping over some reasoning steps that would be helpful for the reader – not least if you're going to keep supporters of the droplet theory reading on.

 Instead, you could start by pointing out that this droplet theory is only one possibility, and that there is another one - the aerosol view, and that this alternative/complementary explanation of how Covid-19 is transmitted is absent from such statements, despite there being solid studies, etc. This is a valid and trustworthy starting point to wonder why another explanation would be left out. Indeed, if one of them contradicts the other or is not supported, then it is understandable. Still, if both are true, it becomes a health policy problem - that we will take decisions that do not address all possible ways that the virus spreads.

 My advice here is not to ‘undersell’ or tone down your contribution but to do this without jumping over some argumentative building blocks. In other words, I believe you can make your claims in a way that shows it is warranted that you explore this situation scientifically – and that the aim of the paper is not to show that droplet theory is wrong.

 Finally, the research questions are good but require the reader to be already acquainted with the Bourdieusian vocabulary that you only introduce later in the paper. Could you simplify these questions or reformulate them in lay terms?

*Theory*

 Being reasonably acquainted with Bourdieu’s work, I recognise its complexity and the fact that the different concepts are interrelated. However, it seems that you mainly use the concepts of field, doxa and illusio in your paper. I think you can drop habitus to simplify the exposition and argument a bit. Moreover, I think you should define what a field is and then immediately identify the three relevant fields, as this identification is also derived from Bourdieu’s work. Otherwise, it is a bit confusing why you come back to the notion of field and why you do not give the same space to the other concepts (cf. my point about keeping the number of concepts at a minimum). Then you can move on to explaining the concepts of illusio and doxa, and then what that means re the droplet vs airborne debate. You do not need any data to do this, and this is for me still part of the framing of your argument, which theorises the empirical phenomenon (absence of the airborne explanation from dominant discourses) that you exposed in the introduction. After reading the entire paper, it seems that you claim this is the first part of your results, but I think it is rather your starting point; it cannot be both. In the empirical part, you go more in-depth into how this unfolded in practice and with what consequences. Still, you do this based on the premise that there was an uneven representation of one explanation over the other in public discourse (this is thus not a finding, but a starting point). You can, of course, start your findings by noting that your analysis confirms your initial observation, but this is not the most exciting point of your paper in my perspective.

*Methods*

 The micro and macro-level focus are well explained, and their interconnectedness is well theorised. This is overall an excellent section on which I have no further comments.  

*Cases and Results*

 Overall, I find that shorter descriptions of the cases and better integration of data and theory would make a more compelling contribution. On the one hand, the cases are quite long and contain most of the data yet are presented descriptively rather than analytically (i.e., presenting a reading of the cases through the Bourdieusian concepts). On the other hand, the results are very abstract and theoretical.

 Again, in line with my comment about the theory part (the need to theorise the droplet vs airborne debate), it is OK to say that your analysis confirms them to be two distinct positions and that airborne transmission is the heterodox one, but this is not the most exciting finding you have. You do not need all this data to claim that there appears to be a knowledge-base / a scientific field constructed as being non-valid, as being out of the doxa. Instead, thanks to your analysis, you can unpack the different cases, how this happens, and how this means interactions between actors in different fields, etc. The same goes for the starting paragraphs about Scientific capital and illusion. Again, you do not need data to make these claims, and you could integrate that in your theoretical framework section. Finally, I think you may want to give a bit more space to the emerging challenges because I found this very interesting in your paper – that there are interstices for change, that the heterodox knowledge can ‘penetrate’ the doxa by specific mechanisms. Also, tell us how this can be explained within your Bourdieusian framework, how this may affect the game, what happens next, etc.

*Discussion*

 The discussion is overall well written and makes some additional points. However, I still have a question after reading the paper: what do your findings mean for the ‘game’ and with what (positive and negative) consequences for practice (including policy-making)? Also, maybe you could carve out a bit more clearly how your discussion and recommendations about interdisciplinarity align with your Bourdieusian framework or distinguish better between the discussion of your results and future avenues that you want to start discussing. For example, we know that interdisciplinary research tends to be both more difficult to publish and less cited. I am happy to be convinced by your argumentation. Still, I just want to point out that having plurality (in scientific and political debate) in the sense of ‘opening the doxa’ or making discussions and decision processes more democratic is different from claiming a need for interdisciplinarity. And, again, it is not clear to me how the concern for interdisciplinarity links to Bourdieu’s theory.

 Finally, coming back to the very beginning of the paper, I have a few minor comments to perhaps make your abstract more compelling and informative:

  

- “both a mystery and a scandal”. I guess it is not a mystery, or at least you have a theory about it! Scandal: it is a scandal once you have answered your research question, not before, right?
- The research question presented in the abstract already contains the answer. In line with some of my other comments, you have to show the public discourse is partial and partisan with your analysis. The only thing you can observe as a starting point is the absence of the airborne explanation from dominant public discourse. So, I would perhaps propose a broader RQ, such as: Why was evidence for airborne transmission side-lined in decision-making regarding infection control policies?
- Methods. Clarify that you are not looking at country cases but cases in different countries. Also, it is not completely clear what difference there is between the theorisation and the application of the theories (operationalisation).
- Results. Political, state and scientific field >> I guess that is part of the theory, rather than a finding. Alignment at all levels with medical scientific doxa >> I think that is more the results. The droplet vs aerosol >> cf. my comments it is the point of departure that they are two distinct approaches, that you then theorise being the orthodoxy vs heterodoxy. How this played out in practice is the exciting part.

Is the work clearly and accurately presented and does it cite the current literature?

Yes

If applicable, is the statistical analysis and its interpretation appropriate?

Not applicable

Are all the source data underlying the results available to ensure full reproducibility?

Partly

Is the study design appropriate and is the work technically sound?

Yes

Are the conclusions drawn adequately supported by the results?

Partly

Are sufficient details of methods and analysis provided to allow replication by others?

Yes

Reviewer Expertise:

Diversity, identity, networks, organisational sociology

I confirm that I have read this submission and believe that I have an appropriate level of expertise to confirm that it is of an acceptable scientific standard, however I have significant reservations, as outlined above.

# Response

**Published:** 2021-07-23

**Authors:** Trish Greenhalgh

**Affiliations:** Nuffield Department of Primary Care Health Sciences, University of Oxford, UK

**Response to reviewer 2 (Florence Villesèche):**

 Thanks for this helpful review. We’ve extensively amended the paper in response.

 We accept the criticism that our commitment to the issue occasionally led us to “make conclusions before providing the evidence”. The revision is more circumspect in tone—mostly by changing the nuance of the argument throughout and emphasising alternative explanations. We explain the detailed changes below.

 We’ve corrected typos – thanks for pointing these out.

 We’ve softened the introductory claim from “incorrect” to “scientifically questionable (and in our view incorrect)”. We’re not presenting the arguments for airborne transmission in this paper; we’re taking them as given and analysing the sociology of the scientific arguments. So we disagree that we need to “build up to” that as our “ultimate” position. It is our starting point. We also state in the very next paragraph that we fully accept that the science is contested. However, we do agree that the statement as initially worded was too blunt. Extraordinarily, there is no scientific evidence that droplet transmission contributes significantly to the spread of Covid-19. The waters have been muddied by the assumption that close-contact transmission was wrongly assumed to be due to droplets.  As the reviewer acknowledges, we don’t really want to get into the science of this here (we’ve argued it extensively elsewhere and we reference those papers), so we have simply softened how we present the term “incorrect”.

 We agree with the reviewer that we could drop much of the discussion on habitus to simplify the line of argument. We have done this in the revised version. We’ve kept in one paragraph where we explain this as a key Bourdieusian concept but removed the sections where we attempt to theorise the habitus of actual individuals.

 We did consider moving the first part of the results to the introduction and including it as our starting point, as this reviewer suggested. But this didn’t work because the description of the orthodoxy and the heterodoxy are *findings*. It took a great deal of analysing texts to draw out the assumptions and lines of argument in the ‘Orthodoxy and heterodoxy’ subsection. We agree however that we didn’t make this clear in the original submission so have added a sentence to explain that these are findings, not background.

 The reviewer suggests shortening the case studies. We tried but found it difficult as we lost nuance. Instead, we’ve deleted one entire case study (USA) to shorten the results section as this focused on a commercial organisation rather than public health bodies which was our main focus. We’ve reduced the length of the other three case studies as much as we can.

 In the Discussion, we’ve highlighted more clearly what our findings mean for the ‘game’ and with what (positive and negative) consequences for practice (including policy-making). We’ve also better aligned our discussion on interdisciplinarity with our Bourdieusian framework.

 We’ve amended the abstract slightly in response to the helpful comments, but we’d like to respectfully push back on this part of the reviewer’s feedback. We would like to keep the word “scandal”. We know it’s an emotive term but we present it clearly as our starting point (the “background” to what we’ve done); we are not pre-empting our findings. In response to the reviewer’s question “it is a scandal once you have answered your research question, not before, right?”, the answer is no, it’s a scandal **before** we answer the research question. The research question is to explore **why** the scandal happened, not to determine whether it was a scandal. We wouldn’t be bothering with this paper if there hadn’t been a scandal to explore. Denial of airborne spread in the face of strong evidence in its favour has quite literally cost—and continues to cost—millions of lives. We agree however that the research question did somewhat pre-empt the findings and have amended as suggested. We also agree that the first sentence of the results in the abstract actually belongs in methods and have moved it.

 Thanks again for this incredibly helpful review.

# Peer review: Reviewer response for version 1

**DOI:** 10.21956/wellcomeopenres.18592.r44065

**Published:** 2021-06-04

**Authors:** Ferric Fang

**Affiliations:** 1 Department of Laboratory Medicine and Pathology, University of Washington, Seattle, WA, USA

The authors may wish to consider revising their article in response to my comments. Otherwise, In my view, the article may be indexed in its present form.

 Among the more important lessons learned during the COVID-19 pandemic has been the importance of airborne transmission, which has had major implications for the prevention of infection by distancing, face mask use, ventilation and the avoidance of crowds. This interesting article discusses the delayed acceptance of airborne transmission by the public health community from a sociological perspective, emphasizing the hegemony of the infectious diseases control field and its initial rejection of dissenting views from experts who primarily came from other scientific fields (e.g., aerosol physics, engineering and chemistry).

 The article illustrates a number of impediments in the way that public policy relating to health matters is guided by science, including tribalism, deference to authority, and dogmatism. It is perhaps an inherent feature of scientific fields to encourage conformity and exclude contributions from outsiders ^1^^,^^2^. A landmark letter published by Morawska and Milton in June 2020 with endorsement by 239 experts called on the medical community and national and international regulatory bodies to acknowledge and address the possibility of airborne SARS-CoV-2 transmission ^3^. The reflexive oppositional alignment of the infection control community is illustrated by the rapid negative response to the Morawska letter, with over 300 signatories insisting that SARS-CoV-2 was mainly spread by “droplets and close contact” and accusing the aerosol scientists of sowing “confusion and fear” ^4^.

 That said, there are limits to framing this debate in purely sociological terms. As a unifying explanation for the dynamic between infection control and aerosol scientists, the Bourdieusian framework falls short. The writers note that Kuhn had little to say about the political dimensions of science. Nevertheless, a Kuhnian perspective would emphasize the relevance of entrenched dogma and the challenge of overturning longstanding paradigms in the case of airborne SARS-CoV-2 transmission. Secondary factors, including political, financial and pragmatic (limitation of available personal protective equipment) considerations, also influenced early messaging relating to transmission and discouraged an emphasis on the possibility of airborne spread. Although infection control specialists may have been misinformed, they considered their views to be evidence-based. They did not reject heterodox views to defend the primacy of their field and defend their “scientific capital,” but rather because they believed their knowledge to be superior and interpreted their observations in the light of an anachronistic paradigm. This meant that some, at least, could eventually be persuaded to change their minds on the basis of new evidence that was inconsistent with spread by direct contact and respiratory droplets. The WHO and CDC now acknowledge that airborne spread of SARS-CoV-2 occurs and can be mitigated by adequate ventilation.

 Some bias in the selection of illustrative case studies is unavoidable. Japan is cited as an exceptional case, in which the infection control field did not dominate the debate. However, Japan was not alone, as a number of Asian countries more quickly accepted the possibility of airborne spread and incorporated face mask use into its pandemic response ^5^. Nor was the public health response a monolithic one, as some infection control experts have cited the precautionary principle, helped to promote more widespread face mask use and sought to obtain more robust evidence of airborne transmission ^6^^,^^7^. As early as April 2020, an advisory report from the National Academies to the U.S. Office of Science and Technology Policy concluded that “the results of available studies are consistent with aerosolization of virus from normal breathing” ^8^. Nevertheless, the dominance of individuals with infectious diseases/epidemiology training and their shared belief in the droplet paradigm was self-reinforcing and led to groupthink in the public health response to the pandemic.

 Another important question raised by this article is the hierarchy of evidence. Evidence-based medicine, which is criticized by these authors, has made an important contribution to improving rigor in the assessment of clinical research. As originally formulated, EBM aspires to integrate systematic research findings with practitioner expertise to guide clinical decision-making ^9^. However, over time the emphasis on randomized clinical trial data as a favoured form of evidence has tended to denigrate the influence of observational data, knowledge of pathophysiology, and expert opinion, which may have delayed the acceptance of airborne transmission of SARS-CoV-2. While randomized clinical trials remain the gold standard for assessing treatment interventions, they are expensive, time-consuming, and ill-suited to determine the route of transmission in the midst of a pandemic caused by a novel pathogen. The limitations of EBM have also been noted in other medical contexts. For example, Tonelli has argued that clinical trials, pathophysiological principles and clinical experience have equal epistemological weight and each warrant consideration in medical decision-making, depending on the circumstances ^10^.

 The conclusions of this article are important. The delayed appreciation of airborne SARS-CoV-2 transmission led to more infections and wasted time and effort on futile preventive measures. It will be important to learn from this experience so that future pandemic responses can consider a broader range of evidence with greater humility and greater acceptance of transdisciplinary input to avoid repeating these mistakes.

Is the work clearly and accurately presented and does it cite the current literature?

Yes

If applicable, is the statistical analysis and its interpretation appropriate?

Not applicable

Are all the source data underlying the results available to ensure full reproducibility?

Yes

Is the study design appropriate and is the work technically sound?

Yes

Are the conclusions drawn adequately supported by the results?

Yes

Are sufficient details of methods and analysis provided to allow replication by others?

Yes

Reviewer Expertise:

Infectious diseases, microbiology

I confirm that I have read this submission and believe that I have an appropriate level of expertise to confirm that it is of an acceptable scientific standard.

## References

[1] : Specialized Science. *Infection and Immunity* .2014;82(4) : 10.1128/IAI.01530-13 1355-1360 10.1128/IAI.01530-13 24421049PMC3993417

[2] : Field Science—the Nature and Utility of Scientific Fields. *mBio* .2015;6(5) : 10.1128/mBio.01259-15 10.1128/mBio.01259-15 PMC460011026350968

[3] : It Is Time to Address Airborne Transmission of Coronavirus Disease 2019 (COVID-19). *Clin Infect Dis.* .2020;71(9) : 10.1093/cid/ciaa939 10.1093/cid/ciaa939 PMC745446932628269

[4] : Airborne Transmission of COVID-19. *Clin Infect Dis* .2020; 10.1093/cid/ciaa1118 10.1093/cid/ciaa1118 32780799PMC7454327

[5] : Rational use of face masks in the COVID-19 pandemic. *The Lancet Respiratory Medicine* .2020;8(5) : 10.1016/S2213-2600(20)30134-X 434-436 10.1016/S2213-2600(20)30134-X 32203710PMC7118603

[6] : A SARS-CoV-2 Cluster in an Acute Care Hospital. *Ann Intern Med* .2021; 10.7326/M20-7567 10.7326/M20-7567 33556277PMC7924623

[7] : Nosocomial outbreak of COVID-19 by possible airborne transmission leading to a superspreading event. *Clin Infect Dis* .2021; 10.1093/cid/ciab313 10.1093/cid/ciab313 33851214PMC8083289

[8] : Rapid Expert Consultation on the Possibility of Bioaerosol Spread of SARS-CoV-2 for the COVID-19 Pandemic (April 1, 2020).2020; 10.17226/25769 10.17226/25769

[9] : Evidence based medicine: what it is and what it isn't. *BMJ* .1996;312(7023) : 10.1136/bmj.312.7023.71 71-72 10.1136/bmj.312.7023.71 PMC23497788555924

[10] : Integrating evidence into clinical practice: an alternative to evidence-based approaches. *J Eval Clin Pract* .2006;12(3) : 10.1111/j.1365-2753.2004.00551.x 248-56 10.1111/j.1365-2753.2004.00551.x 16722902

# Response

**Published:** 2021-07-23

**Authors:** Trish Greenhalgh

**Affiliations:** Nuffield Department of Primary Care Health Sciences, University of Oxford, UK

**Response to reviewer 1 (Ferric Fang):**

 Thanks for this helpful review and suggestions for further references. We’ve included some of the references in the revision.

 We’ve also responded to the comment *“Although infection control specialists may have been misinformed, they considered their views to be evidence-based. They did not reject heterodox views to defend the primacy of their field and defend their ‘scientific capital,’ but rather because they believed their knowledge to be superior and interpreted their observations in the light of an anachronistic paradigm.”  * In the revision, we point out that both Bourdieusian and a Kuhnian analyses assume, broadly speaking, that beliefs within a scientific orthodoxy are honestly held—precisely because they align with prevailing mental models. However, it is also the case that when a mental model is associated with a high degree of scientific capital (that is, when it is associated with power and prestige for the individual or group), the honest scientist will be less inclined to consider changing it. This is precisely the meaning of the term illusio.    

 We agree that Japan is only an example of an Asian country that embraced airborne spread early, and that there are other examples; we’ve noted this in the revised paper. We agree that the infection control field isn’t monolithic. It included a few scientists who did embrace the heterodox position (we have published with some of them!). Nevertheless, infection control *as a sub-field* is currently showing an extraordinary level of recalcitrance which we believe requires scholarly analysis.

 We also agree with the reviewer’s comments about EBM and its hierarchy of evidence—a great deal of good has been achieved but the mental models have become entrenched and over-applied. We’ve published on this previously (1) but don’t want to get distracted into this issue in an already-long paper.

 Thanks again for taking the time to give us such a helpful review. 

 1.         Greenhalgh T. Will COVID-19 be evidence-based medicine’s nemesis? PLOS Medicine. 2020; https://doi.org/10.1371/journal.pmed.1003266.
